# Quantitative Non-canonical Amino Acid Tagging (QuaNCAT) Proteomics Identifies Distinct Patterns of Protein Synthesis Rapidly Induced by Hypertrophic Agents in Cardiomyocytes, Revealing New Aspects of Metabolic Remodeling*[Fn FN2]

**DOI:** 10.1074/mcp.M115.054312

**Published:** 2016-08-09

**Authors:** Rui Liu, Justin W. Kenney, Antigoni Manousopoulou, Harvey E. Johnston, Makoto Kamei, Christopher H. Woelk, Jianling Xie, Michael Schwarzer, Spiros D. Garbis, Christopher G. Proud

**Affiliations:** From the ‡Center for Proteomic Research, Institute for Life Sciences, University of Southampton, Southampton, SO17 1BJ, United Kingdom;; §South Australian Health & Medical Research Institute, North Terrace, Adelaide, SA 5000, Australia;; ¶Clinical and Experimental Sciences Unit, Faculty of Medicine, University of Southampton, Southampton General Hospital, Southampton, UK;; ‖Cancer Sciences Unit, Faculty of Medicine, University of Southampton, Southampton General Hospital, Southampton, UK;; **Department of Cardiovascular Surgery, Jena University Hospital–Friedrich Schiller University of Jena, Erlanger Allee 101, 07747 Jena, Germany;; *^a^* School of Biological Sciences, University of Southampton, Southampton, SO17 1BJ, United Kingdom;; *^b^* School of Biological Sciences, University of Adelaide, Adelaide, SA5005, Australia

## Abstract

Cardiomyocytes undergo growth and remodeling in response to specific pathological or physiological conditions. In the former, myocardial growth is a risk factor for cardiac failure and faster protein synthesis is a major factor driving cardiomyocyte growth. Our goal was to quantify the rapid effects of different pro-hypertrophic stimuli on the synthesis of specific proteins in ARVC and to determine whether such effects are caused by alterations on mRNA abundance or the translation of specific mRNAs. Cardiomyocytes have very low rates of protein synthesis, posing a challenging problem in terms of studying changes in the synthesis of specific proteins, which also applies to other nondividing primary cells. To study the rates of accumulation of specific proteins in these cells, we developed an optimized version of the Quantitative Noncanonical Amino acid Tagging LC/MS proteomic method to label and selectively enrich newly synthesized proteins in these primary cells while eliminating the suppressive effects of pre-existing and highly abundant nonisotope-tagged polypeptides. Our data revealed that a classical pathologic (phenylephrine; PE) and the recently identified insulin stimulus that also contributes to the development of pathological cardiac hypertrophy (insulin), both increased the synthesis of proteins involved in, *e.g.* glycolysis, the Krebs cycle and beta-oxidation, and sarcomeric components. However, insulin increased synthesis of many metabolic enzymes to a greater extent than PE. Using a novel validation method, we confirmed that synthesis of selected candidates is indeed up-regulated by PE and insulin. Synthesis of all proteins studied was up-regulated by signaling through mammalian target of rapamycin complex 1 without changes in their mRNA levels, showing the key importance of translational control in the rapid effects of hypertrophic stimuli. Expression of PKM2 was up-regulated in rat hearts following TAC. This isoform possesses specific regulatory properties, so this finding indicates it may be involved in metabolic remodeling and also serve as a novel candidate biomarker. Levels of translation factor eEF1 also increased during TAC, likely contributing to faster cell mass accumulation. Interestingly those two candidates were not up-regulated in pregnancy or exercise induced CH, indicating PKM2 and eEF1 were pathological CH specific markers. We anticipate that the methodologies described here will be valuable for other researchers studying protein synthesis in primary cells.

Cardiac hypertrophy (CH)^1^ describes the enlargement of the myocardium and can be classified as either the 'physiological' or 'pathological' types. The latter is usually caused by conditions such as chronic hypertension, and is initially an adaptive response to help the heart to maintain normal function. However, sustained pressure overload leads to chronic hypertrophy and myocardial dysfunction. Indeed, pathological cardiac hypertrophy usually progresses into heart failure and is a major cause of death in young adults in developed countries. In contrast, physiological hypertrophy, as caused by pregnancy or exercise, is beneficial (1–4).

The differences between pathological and physiological hypertrophy are profound; for example, fetal genes such as natriuretic peptides A and B are up-regulated in pathological, but not physiological, hypertrophy (5). In pathological hypertrophy, cardiac function usually becomes impaired, whereas in physiological hypertrophy, it is usually preserved or even enhanced. Metabolic remodeling is another significant change during cardiac hypertrophy. In pathological hypertrophy, fatty acid β-oxidation decreases whereas glucose utilization increases. In contrast, both fatty acid oxidation and glucose oxidation are up-regulated in physiological hypertrophy. Pathological hypertrophy is also associated with mitochondrial dysfunction, fibrosis and cell apoptosis; in physiological hypertrophy mitochondrial biogenesis is up-regulated, and neither fibrosis nor apoptosis are observed. Furthermore, distinct signaling pathways are activated in different types of hypertrophy: in physiological hypertrophy, insulin-like growth factor (IGF) 1 activates signaling via phosphoinositide 3-kinase signaling while well-established inducers of pathological hypertrophy, such as the α_1_-adrenergic agonist phenylephrine (PE), which stimulates signaling through G_αq_ and the classical mitogen-activated protein kinase kinase/extracellular signal-regulated kinase (ERK) pathway (6). In both cases, mTORC1 (mammalian target of rapamycin complex 1) signaling is activated. Insulin activates the IGF-1 signaling pathway at the supraphysiological concentration of 100 nmol/L, so was believed to promote the physiological hypertrophic growth of cardiomyocytes at this concentration similarly to IGF-1 (7). However, a recent study showed that insulin signaling actually contributes to the development of pathological cardiac hypertrophy (8). The relationship between pathological CH induced by α_1_-adrenergic stimulation or by insulin has not previously been explored, and doing this is one goal of the present study.

Both pathological and physiological types of CH are a consequence of cardiomyocyte growth. As most of a cell's dry mass is protein, elevated protein synthesis plays a central role in cell growth (9), and mTORC1 is a key positive regulator of protein synthesis and cell growth. Activation of mTORC1 was observed in exercise-induced (10) cardiac hypertrophy. Similarly, in TAC mice, mTORC1 is initially rapidly activated, but subsequently inactivated. Indeed, administration of the mTORC1-specific inhibitor rapamycin prior to or after TAC can attenuate or reverse TAC-induced hypertrophy and heart dysfunction in mice (11, 12). Given the distinct signaling pathways activated α-adrenergic stimulation and insulin in ARVC, and their roles in cardiac hypertrophy, it was therefore important to compare the impact on the synthesis of specific proteins in ARVC.

mTORC1 plays important roles in regulating protein synthesis in isolated adult rat ventricular cardiomyocytes (ARVC). Indeed, activating mTORC1 by over-expressing a small GTPase, Rheb, which positively regulates mTORC1 (13), is sufficient to drive rapid and marked growth of ARVC (14). PE substantially enhances global protein synthesis and ARVC growth, which is largely blocked by rapamycin (14), again indicating that mTORC1 signaling is important for CH. Insulin also stimulates protein synthesis in an mTORC1-dependent manner in ARVC (15).

This study aims to address several key questions: which specific proteins' synthesis is increased in response to different hypertrophic stimuli? Are those proteins also altered in pathological and physiological animal models? Are changes in the synthesis of specific proteins exerted at the level of transcription or translation? Does mTORC1 signaling play a role in regulating the translation of specific mRNAs?

To address these questions, we optimized a recently developed method wherein stable-isotope labeled amino acids are used to tag newly synthesized proteins (pulsed SILAC; pSILAC). Such a pSILAC approach was used to study the regulation of protein accumulation rates by mTOR signaling in HeLa cells (16), a rapidly dividing cancer cell line, which synthesizes proteins at a much higher rate than primary cells such as ARVC. We subsequently improved the pSILAC approach to enhance its selectivity and sensitivity for the analysis of newly synthesized proteins by combining it with azidohomoalanine (AHA) labeling/click-chemistry and subsequent bottom-up LC-MS proteomic analysis (Fig. 1) (17). During incubation in cell culture, AHA (an analog of methionine) becomes incorporated into, and covalently tags, newly synthesized proteins. The resulting azide-derivatized proteins can then be selectively coupled and enriched to biotin-alkyne beads for visualization using fluorescently labeled streptavidin. Alternatively, the azide-derivatized proteins are selectively isolated and enriched with alkyne-functionalized agarose beads and subjected to trypsin proteolysis followed by liquid chromatography-mass spectrometry (LC-MS/MS). The collective analytical attributes of this proteomics approach, referred to as quantitative noncanonical amino acid tagging (QuaNCAT), permits the sensitive and selective measurement of the synthesis rates of specific proteins in cells (18, 19). Other quantitative proteomic approaches that rely on the use of label-free and isobaric stable isotope labeling to examine the differential expression of proteins in hypertrophic or diseased heart (20–22) only capture relative steady-state protein concentration levels rather than assessing rates of *de novo* synthesis. They are thus unsuitable for addressing the aims of the current study. We have further optimized and applied the QuaNCAT approach to measure the regulated synthesis of specific proteins in ARVC cells observed to occur at a very low rate.

**Fig. 1.**
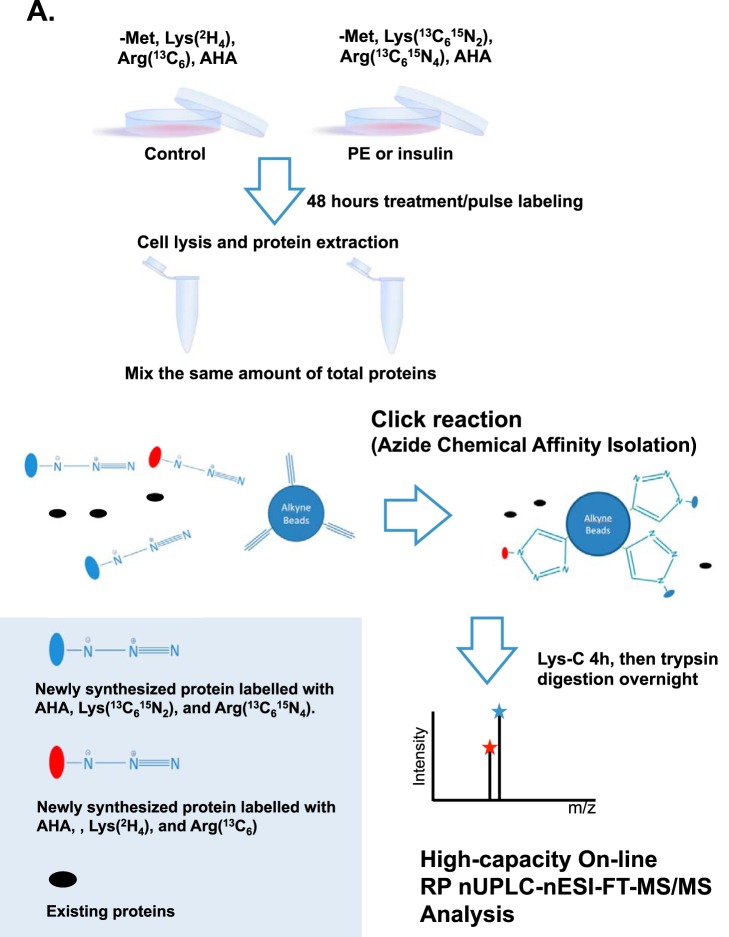
**Scheme of method combining pSILAC with AHA labeling.** See Experimental Procedures for further information.

Our results revealed several key new features in the response of cardiomyocytes to hypertrophic stimuli. These include: (1) both insulin and PE increased the protein synthesis rate to a similar set of proteins. Additionally, marked differences were observed in the patterns of induction of metabolic enzymes and other proteins in response to PE and insulin; (2) increased synthesis of pyruvate kinase M2, a protein involved in anabolic remodeling of cellular metabolism, which may also represent a new early marker for cardiac hypertrophy; (3) the widespread importance of increased translation (rather than transcription) in the rapid effects of hypertrophic agents; and (4) a key role for mTORC1 signaling in the effects of hypertrophic agents of the translation of specific mRNAs, including many mRNAs that are not members of the subset of TOP (terminal oligo-pyrimidine) mRNAs, which are translationally regulated by mTORC1.

## EXPERIMENTAL PROCEDURES

### 

#### 

##### Experimental Design and Statistical Rationale

ARVCs derived from wild-type Sprague-Dawley male rats were treated with phenylephrine (10 μmol/L), insulin (100 nmol/L), and rapamycin (100 nmol/L). AHA-pSILAC was combined with mass spectrometry to identify newly synthesized proteins as a result of each treatment. Replicate experiments were performed, three in the case of insulin and four for phenylephrine.

Identified proteins were validated in ARVCs using two similar but independent methods. We determined the total mRNA levels of selected candidate proteins, to examine whether increased synthesis of these proteins reflects increased translation of their mRNAs or increased mRNA levels.

To test the role of mTORC1 signaling in regulating the synthesis of specific proteins in ARVCs, we repeated the AHA-pSILAC labeling experiments using ARVCs that were treated with PE or insulin in the presence of the mTORC1 inhibitor rapamycin. Finally, key proteomic results were studied using *in vivo* rodent models of pathologic and physiologic cardiac hypertrophy. Data from independent experimental replicates were analyzed using Student's *t* test, Significance was set to *p* = 0.05.

##### Rats

Animals used for isolating ARVC were wild-type Sprague-Dawley male rats from Charles River, Oxford, UK. Animals were reared and sacrificed in line with the United Kingdom Animals (Scientific Procedures) Act, 1986. The method of sacrifice employed here was cervical dislocation according to the UK Home Office regulations Schedule 1. Animals used in the TAC study were as described earlier (23).

##### Isolation of Adult Rat Ventricular Cardiomyocytes

Isolation and maintenance of adult rat ventricular cardiomyocytes (ARVC) were described earlier (15, 24). ARVC were treated with following compounds at the indicated concentrations: phenylephrine (10 μmol/L), insulin (100 nmol/L), and rapamycin (100 nmol/L).

##### pSILAC and Click-It Chemistry Using Invitrogen Alkyne Agarose Resin Beads

ARVCs were cultured in complete M199 medium (M199 medium containing 5.55 mmol/L glucose, 0.68 mmol/L glutamine, 5 mmol/L creatine, 2 mmol/l-carnitine, and 5 mmol/L taurine) after isolation; 30 min prior to any treatments, ARVCs were transferred into Customized M199 media (Dundee Cell Products, Dundee, Scotland) containing 0.41 mmol/L heavy lysine and 0.71 mmol/L heavy arginine, or 0.41 mmol/L medium lysine and 0.71 mmol/L medium arginine. Both media contain low levels of methionine (25.13 μmol/L, about 25% of that in the standard M199 medium). After 30 min, ARVCs were treated with various inhibitors in the presence of 2 mmol/L azidohomoalanine (AHA). Forty-eight hours later, cells were washed twice with cold PBS and lysed in pSILAC lysis buffer (8 mol/L urea, 300 mmol/L Tris-HCl pH 8, 4% (w/v) CHAPS, 1 mol/L NaCI) containing proteinase inhibitors. 400 μg of lysate from the control and treatment animals were mixed together. The mixed protein lysates were reacted with alkyne agarose resin beads. The click reactions were conducted according to the manufacturer's instructions Life Technologies KIT C-10416 Life Technologies, Loughborough, UK, after which, the newly synthesized proteins were covalently conjugated to the alkyne-agarose resin beads. They were then digested using 0.1 μg Lys-C for 4 h, followed by trypsin digestion (0.1 μg/μl X 1 μl 37 °C overnight) and subjected to mass spectrometry analysis, as reported by the authors (17). Replicate experiments were performed, three in the case of insulin and four for PE. This procedure is summarized in Fig. 1.

##### Liquid Chromatography-Mass Spectrometry analysis

Peptide mixtures were de-salted with 100 μl capacity C18 tips (Thermo Scientific) with five incremental iterations from 2 to 98% HPLC grade acetonitrile (Fisher Scientific) in 0.1% analytical grade formic acid (Fisher Scientific). Eluates were lyophilized to dryness and reconstituted in 20 μl of loading solution (2% acetonitrile, 0.5% formic acid). A sample of volume 14 μl was then injected onto a C_18_ μ-Precolumn (300 μm ID × 10 mm L, 5 μm particle; 100 Å pore size, Acclaim PepMap100, Thermo Scientific) at 20 μl/min for 6 min using the loading solution as the mobile phase to trap and de-salt its peptide content. The purified peptides were then loaded onto a high-capacity nano-capillary reverse phase C_18_ column (75 μm × 50 cm, 2 μm particle; 100 Å pore size; Acclaim PepMap 100 column, ThermoScientific) retrofitted to a PicoTip nESI emitter (New Objective, Woburn, MA) and gradient separated as reported by the authors (17). Nanospray ionization was conducted at 2.4 kV and ions were characterized with an FT-Orbitrap Elite (Thermo Scientific) at 240,000 mass resolution. The top 12, +2 and +3 precursor ions per mass spectrometry (MS) scan (minimum intensity 1000) were characterized by high-energy collisional dissociation (HCD; 15,000 mass resolution, 1.2 Da isolation window, 40 keV collision energy) and collision-induced dissociation (CID; ion trap MS, 2 Da isolation window, 35 keV) with a dynamic exclusion (5 ppm) of 200 s.

##### MS Data Processing

Peptide spectrum matching and quantifications were performed with Proteome Discoverer 1.4 (Thermo Scientific) with SequestHT against the UniprotKB SwissProt *Rattus norvegicus* proteome (downloaded 04/2014; 28861 entries). For matching and quantitation, precursor tolerance was set at 10 and 3 ppm, respectively. Fragment matching was set at 0.02 and 0.5 Da for HCD and CID, respectively. Target-decoy searching allowed for 1 missed cleavage, a minimum length of 6 residues and a maximum of three variable (1 equal) modifications of Met-AHA (M), deamidation (Asn, Gln), or phosphorylation (Ser, Thr, or Tyr). Carbamidomethyl (Cys) was set as a fixed modification with Lys (+4 Da), Arg (+6 Da), and Lys (+8 Da), Arg (+10 Da) searched to determine medium and heavy labeled peptides, respectively. The false discovery rate (FDR) was estimated with Percolator at <5% peptide FDR to enable parallel protein grouping and quantitation. The mass spectrometric proteomic data have been deposited to the ProteomeXchange Consortium (25) via the PRIDE partner repository (26, 27) with the data set identifier PXD004127.

##### Protein Hierarchical Clustering Analysis and MetaCore Pathway Analysis

Heat-map construction of proteins analyzed in at least one replicate of all four conditions (*i.e.* Ins *versus* control, PE *versus* control, Ins+Rapa *versus* Ins, PE+Rapa *versus* PE) was generated using Cluster 3.0 (http://bonsai.hgc.jp/∼mdehoon/software/cluster/software.htm) and Java Treeview (http://jtreeview.sourceforge.net). MetaCore (GeneGo, St. Joseph, MI) was applied to identify over-represented biological processes and to identify direct protein interaction networks of proteins of interest. FDR-corrected *p values* < 0.05 were considered significant.

##### pSILAC Plus Click Chemistry Using Invitrogen Alkyne Biotin

ARVCs were cultured and treated as described above, and then lysed in cell lysis buffer (1% Triton® X100, 50 mmol/L β-glycerophosphate, 1 mmol/L EDTA, 1 mmol/L EGTA, 0.5 mmol/L Na_3_VO_4_, 1 mmol/l-dithiothreitol, and proteinase inhibitor mixture; Roche, catalog number 11873580001). Protein concentrations were determined by Bradford assay. Fifty to 200 μg newly synthesized proteins were reacted with biotin-alkyne, according to the procedure described in detail in the manufacturer's instructions (Life Technologies: C-10276). Forty microliters of click reaction products were subjected to SDS-PAGE, then transferred onto a nitrocellulose microporous membrane, which was blocked with 5% (w/v) fat-free powdered milk in PBS-0.02% (v/v) Tween. After three washes with PBS-0.02% (v/v) Tween, the membrane was incubated with streptavidin, Alexa Fluor® 680 (1:10,000 dilution; 45 min) and, after another three washes, the membrane was scanned using the Odyssey® Infrared Imaging System.

##### 5′-RACE (Rapid Amplification of cDNA Ends)

One μg of total RNA from rat heart and a gene-specific primer were used to generate the first strand cDNA containing the 5′-UTR and part of the coding region of mRNA, then use RNase H to degrade the mRNA template. Afterward, the first-strand cDNA was ligated with the DNA oligo linker by T4 RNA ligase. The sequence of this DNA oligo is 5′-CGTTTGCGAGCAGCGTGGCA-3′, the 5′-end of the oligo is phosphorylated, and its 3′-OH is blocked to eliminate self-ligation of the linker. Thus, the unknown 5′-UTR can be amplified by primer pairs complementary to the linker and the coding sequence region. In order to achieve better amplification efficiency, nested PCR (two rounds of PCR with different reverse primer) is required. The PCR product was purified using a gel extraction kit and then sent for Sanger sequencing.

##### Measuring Specific Newly Synthesized Proteins by Click Reaction

Isolated ARVC were cultured and treated with insulin/PE as described above. AHA (2 mmol/L) was added into the medium immediately after the treatment; 48 h later, cells were lysed, and endogenous ACO2 was immunoprecipitated (IP'd) from 250 μg of total lysate protein. IPs were divided into two equal portions; one was analyzed by Western blot to verify the IP efficiency, whereas the other as used for verification of the pSILAC data. IPs were subjected to SDS-PAGE, proteins were transferred onto a nitrocellulose membrane and the membrane was blocked with 5% (w/v) fat-free powdered milk in PBS-0.02% (v/v) Tween. After three washes with PBS-0.02% (v/v) Tween, the membrane was incubated with 1x reaction buffer (prepared in accordance with the manual of Click-iT® Protein Enrichment Kit, Life Technologies: C-10416) containing biotin-alkyne overnight. After the click reaction, the reaction solution was discarded, and the membrane was washed three times with PBS-0.02% (v/v) Tween. Finally, the membrane was incubated with Streptavidin, Alexa Fluor® 680 (1 in 20,000 dilution) for 45 min; after another three washes, the membrane was scanned using the Odyssey® Infrared Imaging System.

##### Cell Culture, Cell Lysis, Western Blotting, Immunoprecipitation, Quantitative Real-Time PCR, and Measurement of Protein Synthesis Rates

HEK293 cells were cultured in complete DMEM (Dulbecco's modified Eagle's medium supplemented with 10% (v/v) fetal bovine serum (FBS) and 2 mmol/L l-glutamine. Penicillin G and streptomycin sulfate were also added to the medium to final concentrations of 100 units/ml and 100 μg/ml, respectively). Other procedures have already been described (28).

5′-RACE primers used in this study are listed in supplemental Table S1.

##### Measuring Newly Synthesized Specific Proteins by [^35^S]-Methionine Labeling

Isolated ARVC were cultured and treated with insulin or PE as described above, [^35^S]methionine (Perkin Elmer, Coventry, UK; 10 μCi/ml) was added into the medium; 48 h later, cells were lysed, and endogenous HSP60 was IP'd from 250 μg total lysate. IP's were subjected to SDS-PAGE and Coomassie brilliant blue staining and newly synthesized HSP60 in the dried gel was visualized by autoradiography.

##### Protein Extraction from Tissues

All mouse or rat tissues (20 mg) were frozen and were pulverized in liquid nitrogen with a mortar and pestle, then lysed with 400 μl RIPA buffer (50 mmol/L Tris HCl 7.4, 150 mmol/L NaCl, 1% Triton X-100 1% sodium deoxycholate, 0.1% (w/v) SDS, and proteinase inhibitors (Roche, complete, EDTA-free Protease Inhibitor Mixture; catalog number: 11873580001)). Protein concentrations were determined by the BCA method.

##### RNA Extraction from Tissues

All mouse or rat tissues (20 mg) were homogenized in liquid nitrogen with a mortar and pestle, then lysed with 1 ml TRIzol®, the following steps of RNA extraction can be found in manufacturer's instructions (Life Technologies: 15596–018). qPCR primers used in this study are listed in supplemental Table S2. Rat 18S rRNA primers for qPCR were from Primerdesign, Southampton, UK.

##### Transverse Aortic Constriction (TAC)

Animals, surgical interventions, clinical assessment, and echocardiographic confirmation of cardiac hypertrophy were described as in (23) (total number of rats = 24).

##### Exercise-induced Cardiac Hypertrophy

Animals at 3 weeks of age were randomly assigned to either the training or the control group (sedentary). The rats in the training group were exercised on a treadmill over a period of 10 weeks with 16% incline, a speed of 25 m/min, and four training episodes per week (Monday, Tuesday, Thursday, and Friday). Running times were incrementally increased as follows: 30 min/d in the first week, 45 min/d in the second week, 60 min/d in the third, 75 min/d in the fourth week, 90 min/d in the fifth week, 105 min/d in the sixth week, and 120 min/d in the last 4 weeks. After 2 and 6 weeks and at the end of the training protocol, animals were analyzed for echocardiographic and *in vitro* examination. As reported earlier, this training regime induces cardiac growth, manifested as an increased heart weight/body weight ratio and other parameters associated with CH (29).

##### Experiment with Pregnant Mice

C57Bl6/J mice (females were ∼4 months of age at the time of euthanization; males were 2–6 months of age at time of mating) were mated and females were checked for plugs daily (total mice number = 38). Once they had plugged, female mice were removed to a separate cage; this was considered day 1 of pregnancy. Control mice comprised female mice that plugged but were not pregnant or equivalently aged mice that had not been mated. Mice were euthanized by cervical dislocation at the indicated times postinsemination and their hearts rapidly dissected out. All mice were confirmed pregnant via dissection and visual inspection of the uterus.

##### Determination of Cell Size

ARVC were seeded on laminin-coated glass coverslips and treated with vehicle control or insulin (100 nmol/L) for 48 h. After treatment, cells were washed twice in phosphate buffered saline (PBS, 18912–014, Gibco) and then fixed in 4% formaldehyde (F8775, Sigma-Aldrich) for 15 min. Fixed cells were washed again thrice with PBS and then labeled with 1× Cellmask stain (C10046, Life Technologies) and mounted in Prolong® Gold Antifade Mountant (contains 4′,6-diamidino-2-phenylindole (DAPI), P36935, Life Technologies). Cells were visualized using Leica TCS SP8X/MP microscope equipped with a tuneable white light laser using a 40× oil immersion objective lens (NA = 1.30) numerical aperture objective. The cross-sectional area was estimated at the center plane of the cell. Relative cell size was then determined using the Leica Application Suite X (LAS X) software (version 1.1.0.12420).

##### Statistical Analysis

Data from independent experimental replicates were analyzed using Student's *t* test, Significance was set to *p* = 0.05.

## RESULTS

### 

#### 

##### Proteomics Methodology Optimization

**Fig. 2.**
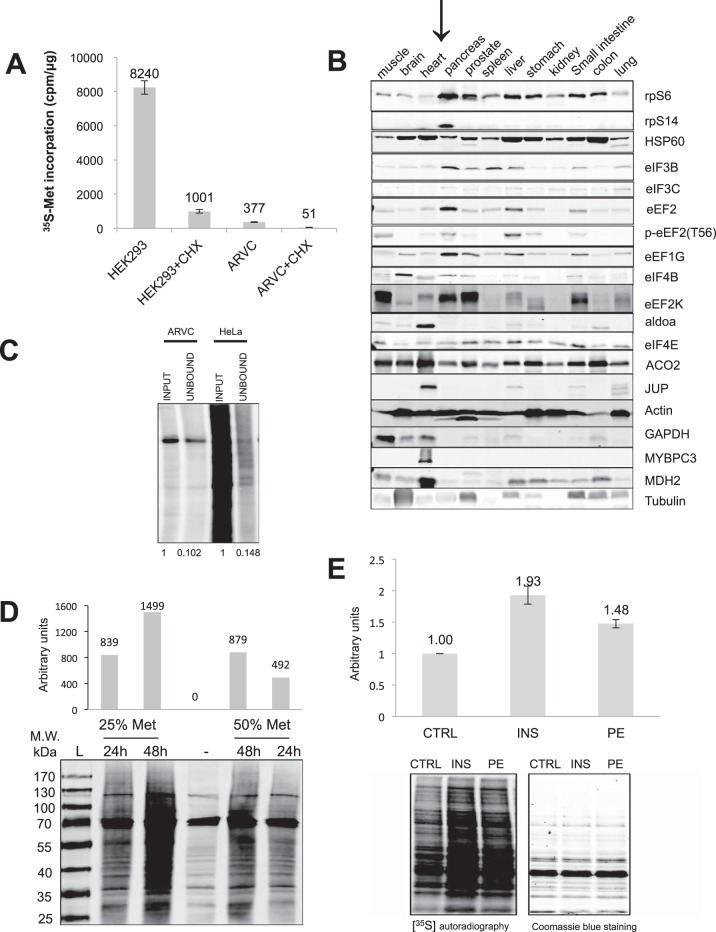
**Optimization of pSILAC conditions.** *A*, ARVC and HEK293 cells were maintained as described in methods; 30 min prior to adding [^35^S]methionine. DMEM was replaced by M199 medium and some cells were treated with cycloheximide (35.54 μmol/L) where indicated. 18 h later, cells were lysed and analyzed for [^35^S]methionine incorporation as described in the Methods. *B*, Twelve different tissues from a single rat were pulverised in liquid nitrogen, then lysed with RIPA buffer. Protein concentrations were measured by the BCA method and 20 μg of protein from each were subject to Western blots. *C*, HeLa cells and ARVC were labeled with AHA for 18 h. Cells were then lysed, and subjected to the 'click' reaction, the alkyne carrier being alkyne-agarose resin. Corresponding amounts of input lysate and supernatant after click reaction were clicked to alkyne-biotin and visualized by Streptavidin, Alexa Fluor® 680. *D*, ARVC were pre-incubated in M199 medium with different concentrations of methionine (25% = 25.13 μmol/L, 50% = 50.26 μmol/L) for 30 min, AHA or normal methionine were then added into the medium immediately after treatment, After different time periods as indicated, cells were lysed, the same amount of total proteins from each group were subjected to click reaction. The alkyne carrier here is biotin-alkyne. After Click reaction, 40 μl of reaction product were analyzed by SDS-PAGE followed by Streptavidin, Alexa Fluor® 680 visualization. The bar graph shows quantification for newly synthesized protein minus negative control (lane 3). *E*, ARVC were incubated in M199 medium with low concentration of methionine (25.13 μmol/L) for 30 min. Some were then stimulated by insulin or PE as indicated and [^35^S]methionine was added to the medium immediately. 48 h later, cells were lysed and 20 μg of total protein were subjected to SDS-PAGE and Coomassie blue staining, whereas newly synthesized proteins in the dried gel were visualized by autoradiography. The bar graph shows quantification for newly synthesized protein:total protein from three independent experiments. Significance was determined by Student's t test.

ARVC do not divide in culture and show low rates of protein synthesis (measured as [^35^S]methionine incorporation), which are only about 5% of the rates in HEK293 cells, a cell line that proliferates rapidly in culture (Fig. 2*A*). Our preliminary data (not shown) indicated that applying the pSILAC approach alone was unable to detect sufficient amounts of labeled peptides with adequate sensitivity because of the masking and/or suppression effects of the highly abundant, nonlabeled background signal from pre-existing proteins. Further investigation showed that levels of ribosomal proteins and translation factors are low in normal rat heart compared with tissues that maintain higher protein synthesis rates such as pancreas or liver (Fig. 2*B*). The AHA-containing newly synthesized proteins generated by our modified approach were captured with ∼90% efficiency by the alkyne agarose resin beads (Fig. 2*C*).

To improve the efficiency of AHA labeling, we placed ARVC in methionine-free medium; this increased the efficiency of [^35^S]methionine incorporation 9-fold (data not shown). However, methionine itself, an essential amino acid, can regulate mTORC1 signaling (30), so we needed to use methionine concentrations that allowed better AHA incorporation efficiency without affecting the mTORC1 signaling pathway. Decreasing the concentration of unlabeled methionine in the M199 medium from 50% to 25% of the standard one also doubled the signal (Fig. 2*D*). Increasing the labeling time from 24 h to 48 h resulted in approximately a doubling of the signals (Fig. 2*D*). Mass spectrometric data (not shown) showed that 48 h labeling-time and a methionine concentration of 25.13 μmol/L in M199 medium provide good conditions to use for ARVC (and increased the number of proteins identified at 24 h from 105 to 167, not shown).

ARVC are particularly challenging cells in which to study protein synthesis because of their very low intrinsic rates of protein synthesis (as documented in this report). This is true whether one uses radiolabeled amino acids (which we have utilized previously, *e.g.* (15)) or stable isotope labeling methods (as used here). In such primary cells, to “scale” this kind of experiments to the desired objectives, [^35^S]methionine incorporation should be performed prior to experiments using AHA labeling. If the rate of protein synthesis in the target cells is similar to cells such as HeLa, 6 h pulse labeling is sufficient to identify 300–400 proteins (16). On the other hand, if the protein synthesis rate in the cells of interest were only 5% compared with HeLa (as it is for ARVCs), then a substantially longer time of labeling would be required (to identify a similar number of proteins, it is theoretically necessary to use a 20-times longer time incubation period). In this case, we labeled for 48 h, the longest feasible time (because, as already noted, ARVCs can only be maintained in culture for a limited period). To help overcome this limitation, we combined pulsed SILAC with AHA labeling to permit us to purify newly made proteins and remove the un-labeled proteins that generate peptides that coelute with, suppress, and mask the presence of labeled proteotypic peptides of interest. For each biological replicate, 0.9–1.2 million ARVCs were used.

To bypass the need to use offline liquid chromatographic peptide purification and separation while preserving proteome coverage, the peptides were on-line de-salted and concentrated with a high-capacity C_18_ μ-Precolumn (300 μm × 10 mm, 5 μm particle; 100 Å pore size) and then loaded onto the 50-cm length reverse phase C_18_ ultraperformance nano-capillary column as reported by the authors (17). Such an on-line chromatographic configuration exhibited the satisfactory loading capacity and separation efficiency needed to cope with the 400 μg total protein, which was injected for each biological replicate run. This approach was, in part, originally developed and applied by Thakur *et al.* (31) for the deep and sensitive proteomic analysis of yeast cell lysates in a single 8-h LC-MS analysis run.

Over 48 h, PE and insulin increased the overall protein synthesis rates in ARVC roughly 1.5-fold and 1.9-fold (Fig. 2*E*), respectively, in line with our previous data (32). Moreover, the intensities of all the visible radiolabeled bands were increased by insulin and by PE (Fig. 2*E*). [^35^S]Methionine incorporation measures global changes in protein synthesis rates, but cannot easily be used to study changes in the synthesis of different protein species (Fig. 2*E*), whereas our new method does readily allow us to identify and quantify changes in rates of synthesis of specific proteins (Fig. 1).

##### PE and Insulin Each Increase the Synthesis of Many Proteins in ARVC

Our previous work showed that 48 h PE treatment promotes hypertrophic growth of ARVC *in vitro*. A recent study showed that excessive activation of the insulin signaling pathway contributes to the development of pathologic cardiac hypertrophy (8). In the present study, we measured the size of ARVC after insulin treatment (100 nm). The data show that 48 h of insulin treatment significantly increases the cells' size (supplemental Fig. S1*A*, S1*B*). Insulin treatment increased the levels of expression of the hypertrophy-associated genes for ANF and BNP (supplemental Fig. S1*C*). Our data support the conclusion that high-dose insulin treatment causes hypertrophic growth of ARVC that resembles more the pathological type. However, insulin does elicit different responses from PE; for example, PE activates the MEK-ERK signaling pathway, which has been shown to play an important role in overload-induced CH (15, 33, 34). On the other hand, insulin activates the PI3K-AKT-mTORC1 signaling pathway (15).

**Fig. 3.**
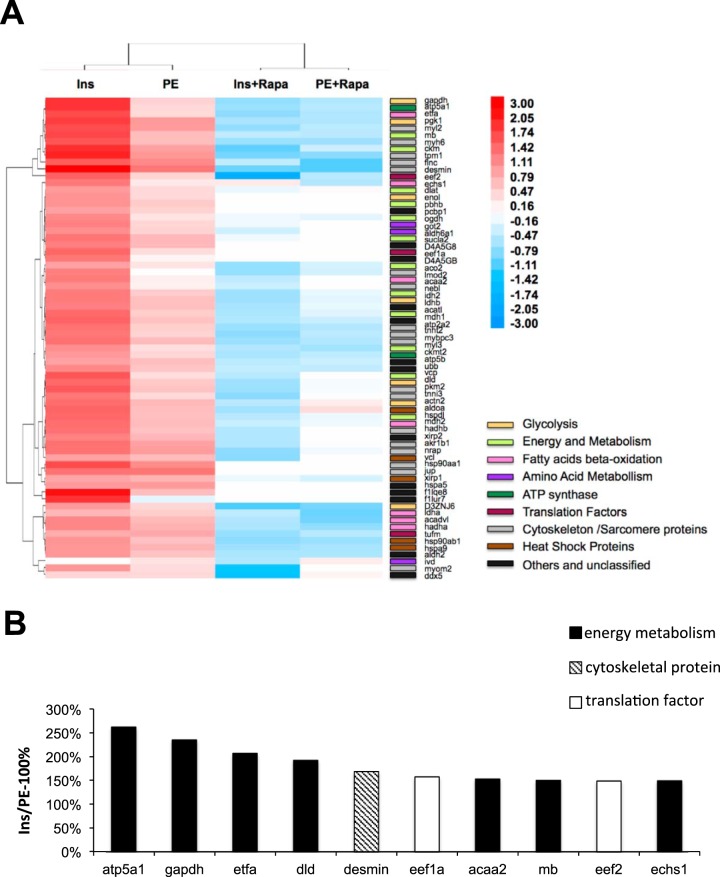
**Hierarchical clustering analysis in heat map format of analyzed proteins.** *A*, Insulin and PE increase the synthesis of a range of proteins via the mTORC1 pathway, as their synthesis is inhibited by rapamycin. The molecular function gene ontology of these proteins were manually curated with ExPASy bioinformatics resource portal (http://www.expasy.org) and mapped to glycolysis, energy and metabolism, fatty acids beta-oxidation, amino acid metabolism, ATP synthesis, translation factors, cytoskeleton/sarcomere proteins, heat shock proteins or other/unclassified proteins. *B*, The ten proteins whose synthesis is increased most strongly by insulin in comparison to PE. The data are plotted as (change in synthesis caused by insulin- change caused by PE)/change caused by PE as a percentage.

The LC-MS data (supplemental Tables S3–S17) show that treatment with PE or insulin increases the synthesis of various categories of proteins including enzymes of glycolysis, the Krebs cycle and fatty acid β-oxidation as well as proteins involved in the cytoskeleton and the contractile machinery. Consistent with the fact that both stimuli cause growth of ARVC and are involved in cardiac hypertrophy *in vivo*, there is considerable qualitative overlap between the sets of proteins, which are up-regulated by the two stimuli. As a means to verify the LC/MS/MS analysis efficiency, the signal response to all proteotypic peptides listed in the reported proteins/peptide lists gave an analytical confidence > 95%. Close inspection of the precursor and product ion spectra of proteotypic peptides exhibited satisfactory signal-to-noise ratios with no appearance of low or saturated ion signal responses. Furthermore, *in silico* Pathway Map analysis (using MetaCore), necessarily restricted only to those proteins identified with an altered synthesis rate (Fig. 3*A*, supplemental Tables S3 and S18), against the entire rat genome confirmed that glycolysis and gluconeogenesis (FDR (False Discovery Rate)-corrected *p* value = 4.4e-5), mitochondrial long chain fatty acid β-oxidation (FDR-corrected *p* value = 7.7e-5), muscle contraction/GPCRs in the regulation of smooth muscle tone (FDR-corrected *p* value = 4.1e-3), and muscle contraction/regulation of eNOS activity in cardiomyocytes (FDR-corrected *p* value = 1.3e-2), were significantly over-represented biological processes. We recognize that this analysis is limited by the fact that even this enhanced QuanCAT method cannot identify and quantify all the proteins whose synthesis rates is changed.

**Fig. 4.**
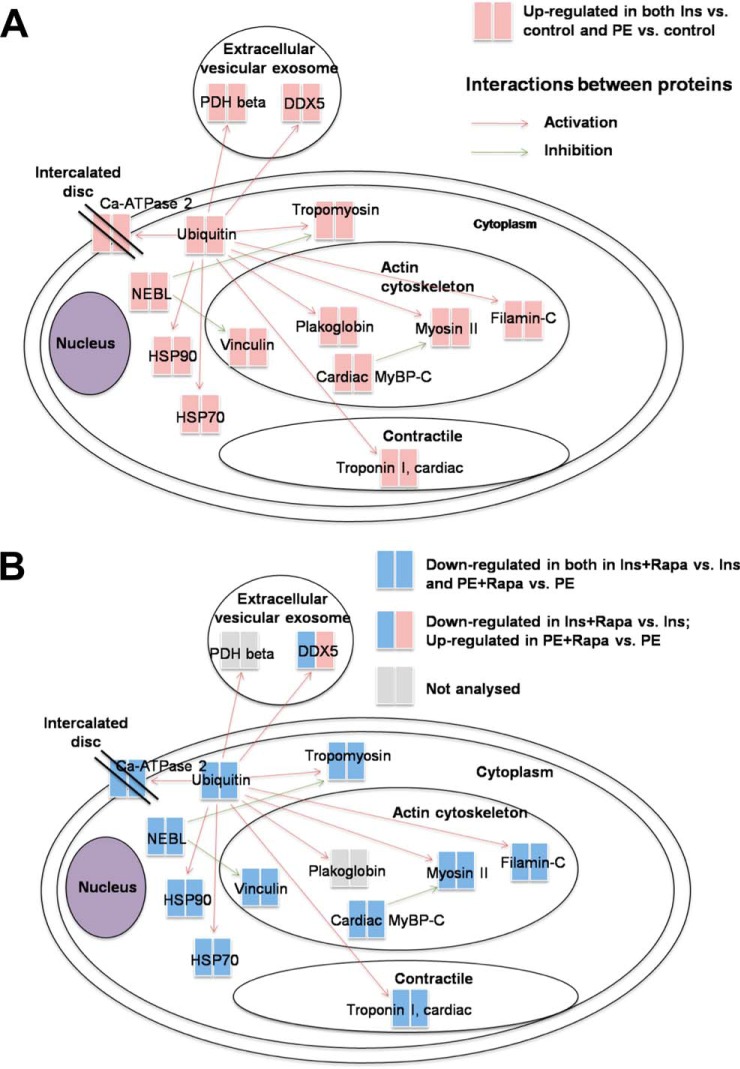
*A*, Direct Protein Interaction Network (PIN) *in silico* analysis of all differentially expressed proteins after insulin and PE activation (*B*) PIN *in silico* analysis of all differentially expressed proteins after insulin + rapamycin and PE + rapamycin activation. PIN analysis was performed with MetaCore and curated with DAVID gene ontologies of subcellular localization. The analyzed proteins are color-coded as red and blue, indicating their up- or down-regulation, respectively. The red, blue and gray lines denote a positive, negative or unspecified regulation, respectively.

Insulin and PE promote the synthesis rates of similar sets of proteins. However, for some proteins the effect of insulin is stronger than PE, for example, insulin increases the synthesis rate of Atp5a1 to 504% and PE increases the synthesis rate of Atp5a1 only to 139%, so the effect of Insulin in the synthesis of atp5a1 is much stronger than PE, then we list top ten proteins in this category whose synthesis was more strongly increased by insulin than by PE (Fig. 3*B*). The data are plotted as (change in synthesis caused by insulin- change caused by PE)/change caused by PE as a percentage. Those proteins are linked to the biological functions energy metabolism (7 proteins), the cytoskeleton (1 protein), or mRNA translation (2 proteins). All these functions appear relevant for the healthy growth of ARVC, *e.g.* during compensated hypertrophy. A direct interaction network of all proteins whose synthesis was found to be differentially regulated after PE or insulin treatment illustrates which of these proteins are functionally interlinked (positive or negative regulation, Fig. 4).

Three important points emerge from the data in supplemental Table S3 and the first two columns of Fig. 3*A*. First, insulin and PE each increase the synthesis of almost all the identified proteins. Interestingly, in almost all cases, insulin increases the synthesis of individual proteins more than PE in line with its greater effect on overall protein synthesis (Fig. 2*E*). Second, the effect of insulin on synthesis of certain metabolic enzymes is greater than its effect on general protein synthesis. Third, the quantitative differences between insulin and PE differ across most protein categories, some being increased by insulin much more than others, relative to PE, *e.g.* Dld (dihydrolipoamide dehydrogenase, required for mitochondrial energy metabolism), Etfa (electron transfer flavoprotein, α-polypeptide, mediates the initial step in mitochondrial oxidation of fatty acids), GAPDH (now known to catalyze a limiting step in glycolysis, (2)) and Atp5a1 (a subunit of the mitochondrial ATP synthase) (Fig. 3*B*). Indeed, seven of the top ten proteins in this group are enzymes involved in energy metabolism, *i.e.* the Krebs cycle, glycolysis or β-oxidation. In particular, GAPDH, which is now known to be the regulatory enzyme of glycolysis under aerobic conditions (35), was much more strongly increased by insulin than by PE (Fig. 3*A*, 3*B*). These changes likely promote a more robust and resilient metabolic profile in insulin-induced hypertrophy. In contrast, the differences between the effects of PE and insulin on synthesis rates are almost nonexistent for most heat shock proteins and less pronounced for many cytoskeletal components (supplemental Tables S3–S17). These quantitative differences for different proteins indicate the existence of clear differences between the effects of the two stimuli studied on the synthesis of specific proteins, rather than simply differences in the magnitude of their effects on all proteins across the board. Our data suggest that rapidly after hypertrophic stimulation, the synthesis rates of many metabolic enzymes are increased, likely to support the increased demand for energy. This could help to explain increased cardiac performance in the compensation stage shortly after pressure overload. The quantitative differences between the rapid effects of insulin and PE in cultured ARVC presumably reflect intrinsic differences between these two stimuli rather than differences because of cardiac morphology or overall metabolism.

##### Validation of Altered Synthesis of Candidate Proteins

**Fig. 5.**
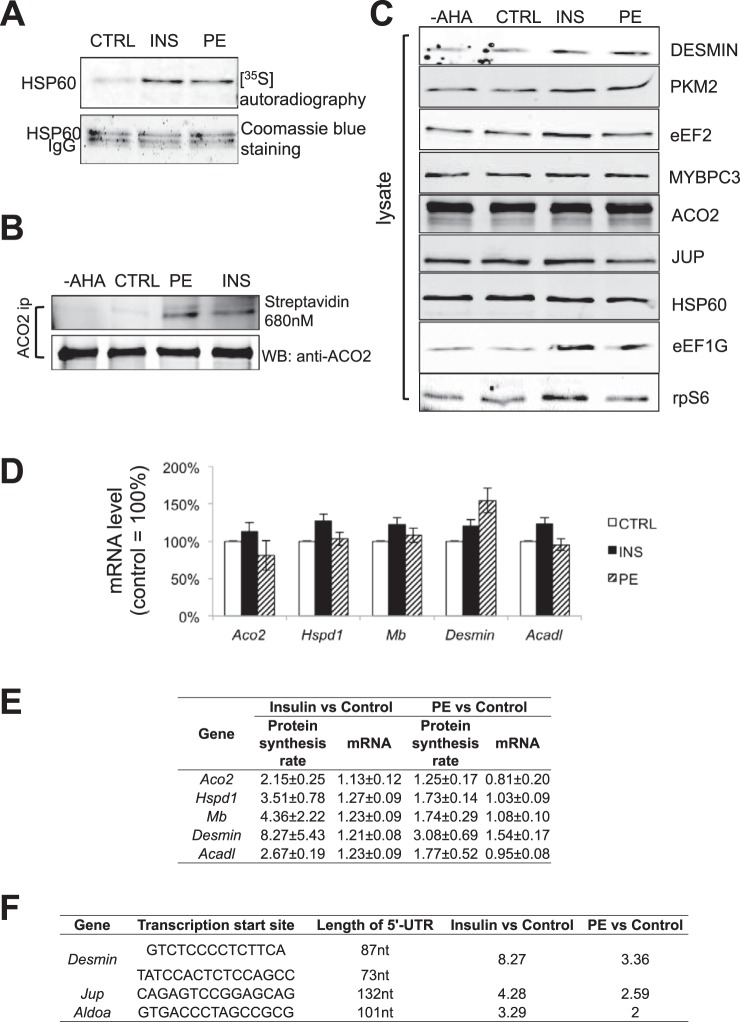
**Validation of pSILAC results in ARVC.** *A*, ARVC were starved in M199 medium containing low methionine (25.13 μmol/L) for 30 min. Cells were then stimulated with insulin or PE as indicated. [^35^S]methionine was added into the medium immediately after treatment and, 48 h later, cells were lysed and HSP60 was IP'd from 250 μg total lysate. IPs were subjected to SDS-PAGE and Coomassie brilliant blue staining, whereas newly synthesized HSP60 in the dried gel was visualized by autoradiography. *B*, ARVC were treated and lysed as in panel *A*. Endogenous ACO2 was then IP'd from 250 μg of cell lysate. IP's were divided into two equal portions; one was analyzed by Western blot to verify the IP efficiency, whereas the other was used for verification of pSILAC result by Click reaction as described in Methods. *C*, ARVC were isolated and maintained as described in Methods. Cells were transferred to low-methionine medium (25.13 μmol/L) 30 min prior to treatment. ARVC were treated with PE or insulin where indicated. Normal methionine or AHA was added into the medium immediately after treatment; 48 h later, cells were lysed and the same amounts of total protein were subjected to Western blots. *D*, ARVC were isolated, maintained and treated as in panel *C*, 48 h later, cells were lysed, total RNA was extracted and subjected to RT-qPCR for the *Aco2*, *Hspd1*, *Mb*, *Desmin*, and *Acadl* mRNAs. *E*, Summary depiction of changes in protein synthesis rates and mRNA levels of selected candidate genes. *F*, 5′-UTRs of the *Desmin*, *Aldoa*, and *Jup* mRNAs.

To validate increased rates of synthesis of specific proteins, we first employed the “traditional” method of [^35^S]methionine labeling, followed by IP with a specific antibody and autoradiography. This method depends upon having an antibody that works well in IP. We tested >10 different antibodies against various candidate proteins, and found, for example, that an HSP60 (heat shock 60 kDa protein 1) antibody was suitable. Our data show that the accumulation rates of HSP60 are indeed increased by insulin and PE in ARVC (Fig. 5*A*). However, the total levels of HSP60 were not changed by 48 h of insulin or PE treatment (Fig. 5*C*), likely because ARVC already contain appreciable amounts HSP60 so the increased rate of synthesis over 48 h does not discernibly affect its total levels. *In vivo*, CH occurs over a longer time period, over which levels of these proteins may indeed change significantly.

AHA labeling provides a nonradioactive alternative to [^35^S]methionine incorporation for assessing changes in the synthesis rates of individual proteins. A strategy comprising AHA labeling, IP with a specific antibody and “clicking” to biotin followed by visualization using fluorescently labeled streptavidin can thus be used to validate the accumulation rate of candidate proteins (supplemental Fig. S2). Using this new method, we found that the accumulation rate of ACO2 (aconitase 2), a mitochondrial protein involved in the Krebs cycle, was increased by insulin or PE (Fig. 5*B*).

If some candidate proteins have low initial levels and/or the accumulation rate is very high, one may see a change in total protein levels even after only 48 h. We did indeed observe that, after 48 h labeling, the total level of desmin (as assessed by Western blot) was increased by both insulin and PE. Insulin also modestly but consistently up-regulated the total protein levels of PKM2 and eEF2 (Fig. 5*C*). In heart, the dominant pyruvate kinase isoform is normally PKM1 (36). PKM1 and PKM2 are encoded by distinct mRNAs derived from the same transcript by alternative splicing, which involves polypyrimidine-binding protein 1 (PTBP1), hnRNPA1 and hnRNPA2. The protein levels of PKM1 and PTBP1 in ARVC were not changed after insulin treatment (supplemental Fig. S3*A*).

The translation elongation factor eEF2 is encoded by a TOP mRNA; such mRNAs contain, immediately adjacent to the 5′-terminal cap structure, a terminal tract of oligopyrimidines (TOP) that confers control by mTORC1. mTORC1 promotes the translation of TOP mRNAs. In ARVC, early studies revealed that cardiac hypertrophy is accompanied by increased ribosome levels in the myocardium (37). We used Western blot analysis to examine another protein that was detected in the proteomic analysis, and is encoded by a TOP mRNA (38), *i.e.* eEF1G, part of the complex that regulates elongation factor eEF1A (39) (supplemental Table S3). We found that the level of eEF1G protein was also up-regulated by insulin in ARVC (Fig. 5*C*). Up-regulation of elongation factors by mTORC1 likely contributes to longer-term mass accumulation during cardiac hypertrophy by increasing the cellular capacity for protein synthesis. Levels of the small ribosomal subunit protein S6, encoded by another TOP mRNA, were also slightly increased by insulin (Fig. 5*C*).

##### Increased Synthesis of Specific Proteins Reflects Increased Translation of Their mRNAs, Not Increased mRNA Levels

Increased synthesis of a given protein could reflect enhanced translation of similar levels of mRNA or, especially after periods as long as 48 h (the labeling time used here), higher mRNA levels because of enhanced gene transcription. We therefore also determined, by RT-qPCR, the total mRNA levels of selected candidate proteins: ACO2, HSP60, myoglobin, desmin, and an acyl-coenzyme A dehydrogenase (Acadl). Their mRNA levels were either unchanged or rose much less than the rates of synthesis of the corresponding proteins (Fig. 5*D*, 5*E*). For example, although desmin mRNA levels increased in response to insulin or PE, this effect was much smaller (1.2- or 1.5-fold) than the 8- or >3-fold changes in the synthesis of desmin itself (Fig. 5*E*). Thus, insulin and PE regulate the synthesis of these proteins primarily by increasing the translation of their mRNAs.

##### PE and Insulin Activates Synthesis of Specific Proteins in ARVC via mTORC1

As noted, both PE and insulin activate mTORC1 signaling in ARVC (15, 33, 34), and mTORC1 also plays a key role in cardiac hypertrophy (11, 12, 40, 41). To test the role of mTORC1 signaling in regulating the synthesis of specific proteins in ARVC, we repeated the AHA-pSILAC labeling experiments using ARVC that were treated with PE or insulin in the presence of the mTORC1 inhibitor rapamycin (RAPA). Hierarchical clustering analysis, presented in heat-map format (Fig. 3*A*), revealed distinct ARVC proteome expression profiles for insulin over control and PE over control relative to the insulin + rapamycin over insulin and PE + rapamycin over PE data sets. Interestingly, rapamycin decreased the synthesis rate of almost every protein observed (Fig. 3*A*, supplemental Tables S18–S30). The direct protein interaction network analysis performed for all differentially expressed proteins after insulin/PE treatment *versus* control and insulin/PE +Rapa treatment *versus* control, shows that a number of those proteins are functionally interlinked (positive or negative regulation, Fig. 4*A* and 4*B*).

For insulin, rapamycin generally decreased rates of synthesis by 45–60%. The greatest inhibition (by almost 75%) was seen for eEF2, which is encoded by a TOP mRNA. Rapamycin also strongly inhibited the synthesis of GAPDH, as reported to be controlled by mTORC1 in HeLa cells by the authors (16), and desmin, which showed the greatest increase in response to insulin. Rapamycin also inhibited the PE-induced increases in the synthesis of every protein, but to a lesser extent (typically by 35–50%) than insulin, in line with the fact that PE also stimulates synthesis of these proteins less than insulin. Again, the strongest effect was observed for eEF2, a protein encoded by a TOP mRNA. These data indicate that increased synthesis of all these proteins is promoted by mTORC1 signaling, in line with our earlier observation that mTORC1 plays a substantial role in the control of protein synthesis by PE or insulin in ARVC (32).

##### Determination of the 5′-UTR of Candidate mRNAs

Given mTORC1's major role in regulating synthesis of many proteins at the translational level in ARVC and that mTORC1 promotes translation of TOP mRNAs (42), it was of interest to determine the sequence of the 5′-UTRs of selected candidates. We therefore employed a method that is based on a previously published approach (43). Using this method (supplemental Fig. S4), we determined the sequence of the 5′-UTRs of the mRNAs encoding desmin, ALDOA (aldolase A) and JUP (junction plakoglobin) in ARVC (supplemental Fig. S5, Fig. 5*F*). These are not TOP mRNAs and do not possess the pyrimidine-rich translational element previously reported to play a role in mTORC1-mediated translational control (44). Their 5′-UTRs are actually short and unstructured, so they do not resemble the types of structured mRNAs thought to be controlled by the mTORC1-regulated eIF4F complex. Interestingly, we have previously shown that PE regulates protein synthesis in ARVC by mechanisms that depend on mTORC1 but in an eIF4F-independent manner (45).

##### PKM2, and eEF1G are Up-regulated in TAC Induced Hypertrophy, But Not in Physiological CH Models

**Fig. 6.**
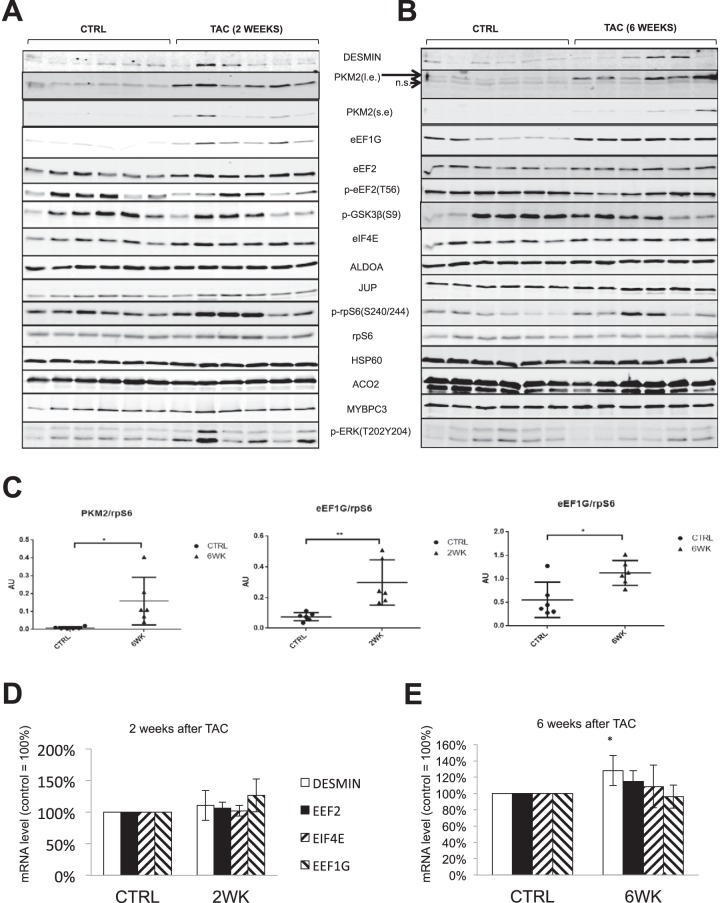
**Expression of PKM2 and eEF1G is up-regulated in TAC animal model.** *A*, *B*, TAC operation was as described in EXPERIMENTAL PROCEDURES. At two- and six-week time points, six experimental rats and six age-paired controls were sacrificed; left ventricles were pulverised in liquid nitrogen, then lysed with RIPA buffer, and protein concentration was measured by the BCA method. 20 μg of protein from each heart was subjected to Western blots. *C*, Quantification of PKM2 and eEF1G from panels *A*, *B. D*, *E*, Control and TAC rats were as described in panel *A* (six in each group). At two- and six-week time points, six experimental rats and six age-paired controls were sacrificed. Total RNA was extracted from the left ventricles by TRIzol and analyzed by RT-qPCR.

TAC (transverse aortic constriction) is a classic model of pathological cardiac hypertrophy caused by pressure overload, and induces increased heart size (relative to body weight and other hallmarks of CH (46). TAC induces activation of mTORC1 (11, 47), which is important in mass accumulation following TAC. Our data show that, 2 weeks after TAC, three out of six rats still exhibited very high levels of mTORC1 signaling (rpS6 p240/244 phosphorylation) (Fig. 6*A*), so it was of interest to study whether candidate proteins identified by pSILAC were also up-regulated in the TAC-rat model.

Expression of PKM2 was elevated following TAC at the 6-week time point (Fig. 6*A*–6*C*), indicating that PKM2 may play important roles in this setting. Expression of eEF1G is up-regulated in the TAC model at both 2- and 6-week time-points. Increased expression of translation factors in the TAC model likely helps support the faster protein synthesis that leads to mass accumulation during cardiac hypertrophy. The mRNA levels for eEF1G in TAC samples were not changed (Fig. 6*D*, 6*E*), indicating its up-regulation is at a translational level. The mRNA for desmin was slightly up-regulated (20%); however, desmin protein levels in TAC samples were unaltered, which differs from what was observed in ARVC in culture. Using two-dimensional electrophoresis (2-DE) and mass spectrometry, it was found that, at 48 weeks, when left ventricular hypertrophy is established, desmin protein levels are up-regulated (48). In order to assess whether PKM2 and eEF1G were also up-regulated in physiological cardiac hypertrophy model, we used Western blot analysis samples from in exercise-induced CH heart (rat), and hearts from pregnant mice, because two independent studies did not see CH in pregnant rats, even though cardiac function was clearly enhanced (49, 50). Our data show that PKM2 and eEF1G were not up-regulated in those two models (supplemental Fig. S6), indicating PKM2 and eEF1G were specifically up-regulated in pathological CH. Interestingly, a recent study reported that PKM2 is also up-regulated in the heart of mice treated with sunitinib, an anti-cancer drug whose use is associated with heart failure (51). We found that protein levels of PKM1 and PTBP1 in TAC samples were not changed (supplemental Fig. S3*B*, S3*C*).

##### Chronic Activation of mTORC1 in MEFs Up-regulates the Expression of Candidate Proteins

**Fig. 7.**
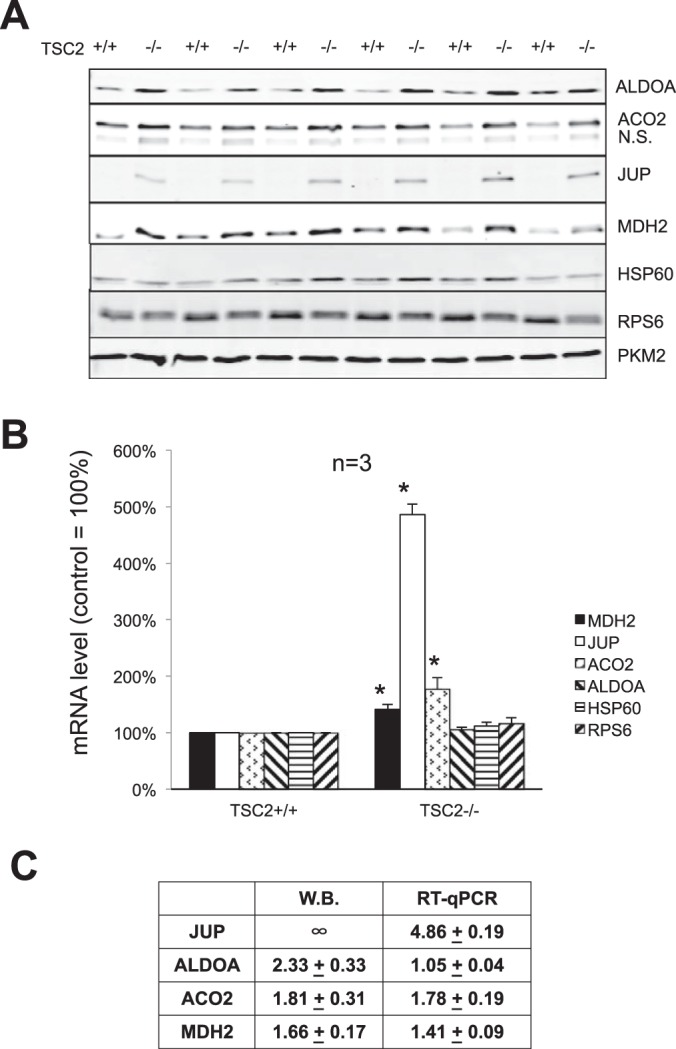
**Chronic activation of mTORC1 in MEFs increases the expression of JUP, ADLOA, ACO2, and MDH2 proteins.** Wild-type and TSC2^−/−^ MEFs were cultured in complete DMEM. *A*, After lysis, 20 μg of total protein were subjected to Western blot using the indicated antibodies. *B*, After lysis, total RNA was extracted and subjected to RT-qPCR analysis for *Mdh2*, *Jup*, *Aco2*, *Aldoa*, *Hspd1*, and *Rps6. C*, Quantification and summary table of panels *A* and *B*.

For candidate proteins whose synthesis is regulated by mTORC1 signaling in ARVC, chronic activation of mTORC1 is expected to upregulate their total levels. Because loss of TSC2, a negative regulator of mTORC1, leads to constitutive activation of mTORC1 (52), we determined the total protein and mRNA levels for several candidates in wild-type and TSC2^−/−^ MEFs (Fig. 7). This will tell us whether the candidates identified in ARVC are heart cell-specific or universal.

The results clearly show that the protein levels of ALDOA, ACO2, JUP, and MDH2 are elevated in TSC2^−/−^ MEFs compared with wild-type MEFs, especially for JUP, which was not detectable in wild type MEFs, but expressed at substantial levels in TSC2^−/−^ MEFs (Fig. 7*A*). The *Jup* mRNA level was also increased by almost 4-fold in TSC2^−/−^ MEFs. The mRNA for ALDOA was not affected by activation of mTORC1, but its protein levels in TSC2^−/−^ MEFs are about 2.3-fold higher than in wild-type MEFs (Fig. 7*B*, 7*C*). These results indicate that mTORC1 regulates the expression of ALDOA at the translational level. In MEFs, expression of MDH2 and ACO2 were also up-regulated by mTORC1, but are probably regulated at the transcriptional level in MEFs, which differs from the situation observed in ARVC (Fig. 5*E*).

## DISCUSSION

Here, we report the further optimization, refinement and novel application of a recently developed QuaNCAT proteomics approach (19, 53) to analyze the synthesis rates of specific proteins in response to stimulation of ARVC with pro-hypertrophic agents. This approach provides an unbiased and effective means to quantify changes in the synthesis of many proteins in parallel, even in cells with low ongoing rates of protein synthesis. We have also developed a new and effective method for validating changes in the synthesis of specific proteins identified using this approach. Both of these approaches will be valuable for studying changes in the synthesis of specific proteins in a wide range of cell-types and conditions.

Our QuaNCAT proteomics data reveal that, in response to hypertrophic stimulation, the synthesis rates of many proteins increase markedly even over the first 48 h. Insulin and PE each increase the synthesis of a wide range of proteins. According to the prior literature, the total levels of some of these proteins are also up-regulated at later stages of pathological CH, *e.g.* the sarco/endoplasmic reticulum Ca^2+^-ATPase, GAPDH, myoglobin, mitochondrial elongation factor EF-Tu (Tufm), 2-oxoglutarate dehydrogenase and phosphoglycerate kinase, JUP (Junction Plakoglobin), VCL (Vinculin), HSP90aa1, HSP90ab1, Ddx5, eEF1A1, eEF2 (54), filamin-C (34, 54), and ENO1 (20, 54). The accordance between the current study and previous ones demonstrates the validity of our newly developed method. Our data also show the elevated synthesis of those proteins in CH is mediated primarily at the level of the increased translation of their mRNAs driven by mTORC1 signaling. Thus, activation of the synthesis of specific proteins is a very rapid translational response to hypertrophic simulation of ARVC, which precedes the long-established but slower increase in their translational capacity (ribosomal content) (2).

Our data also show that PE and insulin promote the synthesis of generally similar sets of proteins. However, on average, the increase in their rates of synthesis was 102% greater for insulin than PE. For certain specific proteins, the relative rate of stimulation between the insulin and PE was found to be even larger (Fig. 3*A*); namely, among the top ten proteins in this category, seven were metabolic enzymes, and only one was a structural cytoskeletal protein. The differential increase in the rate of synthesis of these two groups of proteins is likely to give rise to different metabolic outcomes in the two types of settings, because insulin more strongly enhances the expression of proteins involved in supplying metabolic energy, which is required for enhanced cardiac function.

As stated, GAPDH is known to be a rate-limiting enzyme for aerobic glycolysis (35). Another essential participant to aerobic glycolysis is pyruvate kinase, which catalyzes the transformation of phosphoenolpyruvate into pyruvate and exists as four isoforms, PKL, PKR, PKM1, and PKM2. In adult animals, the dominant form of PK in heart is PKM1 (36). Given the role of PKM2 in regulating the channeling of carbon atoms into biosynthesis or oxidation (55), the ability of PE and especially insulin to increase the synthesis of GAPDH and PKM2 is of particular interest. Interestingly, we found that the synthesis of PKM2 is up-regulated by PE/insulin via mTORC1 signaling in ARVC. The levels of PKM2 protein are also up-regulated in hearts from animals subjected to TAC. The biological significance of PKM2 is underscored by the observation that it plays important roles in tumorigenesis (56). In various types of tumors, pyruvate kinase expression shifts to the PKM2 isoform (57). Cancer cells make use of anaerobic glycolysis (58) to utilize glucose efficiently to generate biomass (the “Warburg effect”), *i.e.* the synthesis of macromolecules required for cell growth and division. PKM2 plays a key role in regulating the direction of carbon atoms toward biosynthesis or oxidation (55). In TAC rats, upregulating PKM2 likely also serves to provide precursors for macromolecular synthesis to support cardiomyocyte growth.

These changes suggest that the alterations in intermediary metabolism induced by hypertrophic stimuli are similar to those seen in cancer cells, where enhanced metabolic capacity is also a key feature. Additionally, PKM2 is the isoform initially expressed in embryonic tissues, so the increased expression of PKM2 during TAC may reflect the recapitulation of a fetal gene program. We used five pairs of qPCR primers to try to measure *Pkm1* and *Pkm2* mRNA levels in ARVC or rat hearts, but unfortunately, none worked in qPCR, so we were unable to assess the mRNA levels for these two isoforms. Because one of the major proteins involved in the alternative splicing of *Pkm1/2* mRNA and of the PKM1 protein itself are not altered (supplemental Fig. S3), it is unlikely that up-regulation of PKM2 under pressure overload is (solely) mediated by alternative splicing. On the other hand, our data indicate that increased translation of PKM2 mRNA is mediated by mTORC1 signaling. Regardless, other factors may also be involved. Rees *et al.* (51) reported that, in a sunitinib induced heart failure mouse model, the PKM2 protein level was up-regulated by 2-fold, but PKM1 remained unchanged, which is very similar to what we observed in the TAC rat model. They also found shift of about 25% from the PKM1 mRNA to the PKM2 mRNA. This shift was significant but quite subtle compared with the change in protein level (2-fold). Given that sunitinib also increased insulin sensitivity in mice, it is highly likely that insulin signaling pathway and increased translation also contributed to the up-regulation of PKM2 in their model in addition to alternative splicing. Significantly, our data also suggest that inhibiting PKM2 might be a new therapeutic approach for pathological cardiac hypertrophy therefore serving as a potential biomarker of response to pharmacologic intervention. However, although inhibitors of PKM2 such as Shikonin and its analogs have recently been described and shown to inhibit cancer cell glycolysis (59), it is not clear that these compounds are suitable as therapeutic agents for targeting PKM2, because they also interact with PKM1 and other proteins (60).

We also observed increased expression of one elongation factor (eEF1G), a component of the guanine nucleotide that controls the activity of eEF1A, an effect that likely contributes to increasing protein synthesis capacity during pathological CH.

The healthy heart normally prefers fatty acids as its energy source (fatty acid oxidation provides about 70% of the ATP in healthy heart (61). The heart can also use certain other substrates as energy sources, including glucose, lactate, ketones and amino acids (62, 63). Such flexibility can help maintain normal function under different workloads and circumstances. Metabolic remodeling in heart is a hallmark of CH. Under hypertrophic conditions, total ATP production decreases, and the proportion of ATP generated by fatty acid oxidation is reduced, whereas ATP generation by glycolysis and lactate oxidation increases. Interventions that block the substrate shift from fatty acids to glucose can attenuate TAC-induced cardiac hypertrophy (61). Our findings indicate that, following short-term hypertrophic stimulation, the expression of enzymes involved in glycolysis and the Krebs cycle increases, likely as an adaptation to the increased demand in ATP. Although it is commonly accepted that, in failing heart, genes involved in fatty acid β-oxidation are downregulated (64), our data showed that synthesis rate of β-oxidation enzymes is also up-regulated soon after hypertrophic stimulation. Our results suggest that myocytes initially attempt to maintain a high level of β-oxidation after hypertrophic stimulation by upregulating those enzymes. However, in the decompensation stage of CH, impaired mitochondrial biogenesis and mitochondrial defects occur (65), rendering the up-regulation of those enzymes unfeasible. Interestingly, analogous traits have been observed with LC-MS-based quantitative metabolomics studies using a mouse model of dilated cardiomyopathy (66) and rat TAC hearts (67).

Numerous sarcomeric components/cytoskeletal proteins were identified as showing increased synthesis in our pSILAC experiments, several of which play important roles in cardiac function and/or in cardiomyopathies (*e.g.* desmin, vinculin, NRAP (nebulin-related anchoring protein) and Lmod). Mutations or altered expression of those proteins are associated with various cardiac disorders (68–76). Our data show that expression of these proteins is regulated by mTORC1 signaling in ARVC.

**Fig. 8.**
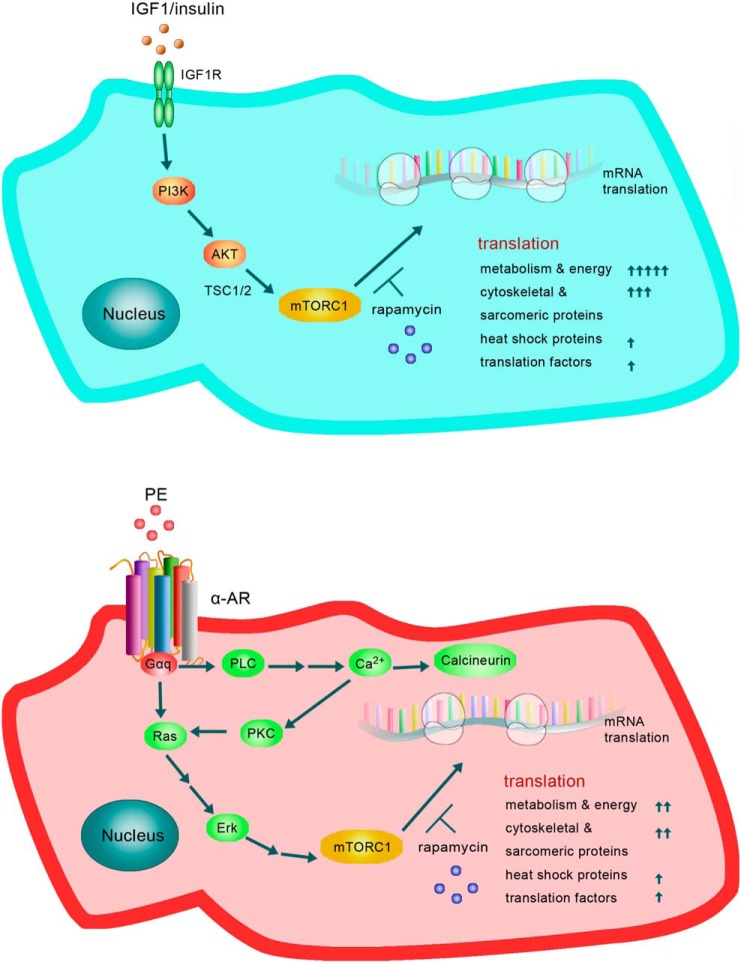
**Metabolic remodelling model reflected in the distinct patterns of protein synthesis induced by the PE and Insulin in cardiomyocytes, (see Discussion for details).**

Indeed, up-regulation of the synthesis of all the proteins we identified was inhibited by rapamycin indicating it requires signaling through mTORC1 (Fig. 8). We determined the levels of the mRNAs for several proteins of interest by RT-qPCR. mRNA levels were either unchanged or minor indicating that enhanced synthesis of these proteins involves increased translation of their mRNAs, rather than increased mRNA levels, consistent with the well-established role of mTORC1 in controlling mRNA translation. Thus, a key feature of the early response of ARVC to hypertrophic stimuli is that translational control underpins the increased synthesis of proteins in response to hypertrophic stimuli in ARVC (Fig. 8). We have previously shown that insulin and PE each activate multiple components of the translational machinery in ARVC (15, 33, 34, 77).

The greatest effect of rapamycin on the synthesis of a specific proteins was observed for eEF2, which is encoded by a TOP mRNA. However, the vast majority of the proteins whose synthesis was sensitive to rapamycin are not known to be encoded by TOP mRNAs. Our novel rapid amplification of cDNA ends (RACE) technique confirmed that the *desmin*, *AldoA,* and *Jup* mRNAs do not contain a TOP. Thus, the synthesis of these proteins is increased via an mTORC1-dependent mechanism distinct from the established TOP regulatory system. Interestingly, some candidate proteins (ALDOA, MDH2, JUP, ACO2) identified in our pSILAC data are not only regulated by mTORC1 signaling in ARVC but also in MEFs. Surprisingly, eEF1G and PKM2 protein levels remained unchanged in TSC2^−/−^ cells *versus* controls (data not shown).

The study results underscore the notion that our optimized QuaNCAT proteomics approach can more effectively quantify proteins with very low synthesis rates commonly occurring in cardiomyocytes. However, a significant number of many proteins were profiled with a high abundance of “light” proteotypic peptides relative to their “medium” or “heavy” counterparts. Although this difference in ion intensities may have reduced the linear dynamic range of relative quantitation, this trend had negligible effects to the study method's ability to profile AHA-pSILAC labeled peptides that corresponded to proteins with low synthesis rates. Such a performance characteristic was extensively verified as part of the method optimization experiments. Our proof-of-concept study was intended to assess changes in the synthesis rates of specific proteins rather than observing the wider spectrum of proteins typically detected under steady-state conditions using nontargeted isobaric labeling approaches or label-free approaches (20–22). Our method selectively analyzed newly synthesized proteins and exhibited a wide linear dynamic range capturing proteins occurring at the higher native abundance levels (*i.e.* cytoskeletal and contractile proteins) that mapped to the canonical pathway analysis performed as well as proteins that occur at the lower native abundance levels (*i.e.* the translation factors eEF1G and eEF2, PKM2) that constitute novel observations of this study and provide additional measurable hallmark features in ARVC. Also, the performance characteristics of our optimized QuaNCAT method in terms of analytical precision, selectivity, accuracy, and sensitivity supersedes that of older radiolabeling approaches (*e.g.* with [^35^S]methionine) for studying the synthesis of specific proteins. There are undoubtedly newly synthesized proteins in ARVC that are not captured with the method used here because of intrinsic limitations in the enrichment, purification and/or subsequent LC-MS detection of peptides. Therefore, the synthesis of other proteins of potential interest likely shows differential responses to PE or insulin. This does not detract from the basic study goal in that measurable and novel differences were indeed observed between the reported stimuli in ARVC. Previous studies using label-free and iTRAQ LC-MS proteomics to study the differential expression of proteins in hypertrophic or diseased heart (20–22) only examined steady-state levels of expression, not alterations in their synthesis rates. Another proteomics study using two-dimensional electrophoresis (2-DE) and MALDI mass spectrometry, found that, at 48 weeks, when left ventricular hypertrophy was well established, 56 proteins were differentially expressed in hearts of spontaneously hypertensive rats compared with controls (48). The same group reported only nine proteins were differentially expressed at earlier stages of left ventricular hypertrophy (22). By contrast, the detection of proteins exhibiting a varying degree of synthesis rates and native abundance levels made possible with the QuaNCAT study method were not observed by these proteomics approaches even at steady-state concentration levels. Additionally, the degree of proteome coverage for the *de novo* synthesized proteins observed in this study mapped to a wide spectrum of biological pathways and networks thus providing a novel synergistic understanding of cardiac hypertrophy.

## CONCLUSIONS AND FUTURE PERSPECTIVES

Overall, our data show that, rapidly after PE or insulin stimulation of ARVC, there are substantial changes in the synthesis rates of proteins involved in various processes including glycolysis, Krebs cycle, β-oxidation, translation, protein folding and sarcomeric function. Their regulation requires mTORC1 signaling, which promotes translation of their mRNAs. Our data thus reveal that ARVCs respond rapidly to hypertrophic stimuli by upregulating the synthesis of proteins involved in metabolism and cytoskeletal/contractile function, although the responses to PE and insulin differ quantitatively for several proteins. The latter aspect may well to a considerable extent lead to the differing eventual outcomes to stimulation of heart muscle cells by PE or insulin. As a future perspective, the PE and insulin conditions, with or without rapamycin, at the reported concentrations levels provide useful new insight in the design of functional ARVC *in vivo* assays to further substantiate these study conclusions. Finally, we show that two of these proteins, PKM2 and eEF1G, are specifically up-regulated in rats subjected to TAC (Fig. 7 and supplemental Fig. S6). Thus, our proof-of-principle study has identified PKM2 as a candidate biomarker of pathological hypertrophy that warrants additional functional validation with commercially available small molecule inhibitors in various *in vivo* models.

## Supplementary Material

Supplemental Data

## Footnotes

^1^ The abbreviations used are:

CH: Cardiac hypertrophy
2-DE: Two-dimensional electrophoresis
4E-BP1: 4E-binding protein 1
Acadl: Acyl-Coenzyme A dehydrogenase, long-chain
ACO2: Aconitase 2
AHA: L-azidohomoalanine
ALDOA: Aldolase A
ARVC: Adult rat ventricular cardiomyocytes
CDS: Coding DNA sequence
FDR: False discovery rate
hnRNPs: Heterogeneous nuclear RNA-binding proteins
HSP60: Heat shock 60kDa protein 1
IGF1: Insulin-like growth factor 1
Lmod: Leiomodin
MDH2: Malate dehydrogenase 2
mTORC1: mammalian target of rapamycin complex 1
NRAP: Nebulin-related anchoring protein
PE: Phenylephrine
QuaNCAT: Quantitative Noncanonical Amino acid Tagging
pSILAC: pulsed stable isotope-labeling with amino acids in cell culture
PTBP1: Polypyrimidine tract-binding protein 1
RPs: Ribosomal proteins
S6Ks: S6 kinases
TAC: Transverse aortic constriction
TOP: Terminal oligopyrimidine tract.
