# Supplementary material for: Quantitative Non-canonical Amino Acid Tagging (QuaNCAT) Proteomics Identifies Distinct Patterns of Protein Synthesis Rapidly Induced by Hypertrophic Agents in Cardiomyocytes, Revealing New Aspects of Metabolic Remodeling
Source: Mol Cell Proteomics. 2016 Aug 9;15(10):3170–89. doi: 10.1074/mcp.M115.054312 (PMC5054342; doi:10.1074/mcp.M115.054312)
Supplement: Supplemental Data [file supp_15_10_3170__index.html]

Quantitative Non-Canonical Amino acid Tagging based proteomics identifies distinct patterns of protein synthesis rapidly induced by hypertrophic agents in cardiomyocytes, revealing new aspects of metabolic remodeling — Quantitative Non-canonical Amino Acid Tagging (QuaNCAT) Proteomics Identifies Distinct Patterns of Protein Synthesis Rapidly Induced by Hypertrophic Agents in Cardiomyocytes, Revealing New Aspects of Metabolic Remodeling — Translational Control by Hypertrophic Agents in Heart Cells — Supplemental Data 

# Quantitative Non-canonical Amino Acid Tagging (QuaNCAT) Proteomics Identifies Distinct Patterns of Protein Synthesis Rapidly Induced by Hypertrophic Agents in Cardiomyocytes, Revealing New Aspects of Metabolic Remodeling

## Supplemental Data

- Supplemental Table S1 (.docx, 15 KB) - Supplemental Table S1
- Supplemental Table S2 (.docx, 18 KB) - Supplemental Table S2
- Supplementary Table S3 (.xlsx, 22 KB) - Summary Table of Ins or PE vs. Ctrl
- Supplementary Table S4 (.xls, 1.7 MB) - Ins vs. Ctrl - Experiment 1 - Protein List
- Supplementary Table S5 (.xlsx, 900 KB) - Ins vs. Ctrl - Experiment 1 - Peptide List
- Supplementary Table S6 (.xls, 1.7 MB) - Ins vs. Ctrl - Experiment 2 - Protein List
- Supplementary Table S7 (.xlsx, 924 KB) - Ins vs. Ctrl - Experiment 2 - Peptide List
- Supplementary Tale S8 (.xls, 473 KB) - Ins vs. Ctrl - Experiment 3 - Protein List
- Supplementary Table S9 (.xlsx, 324 KB) - Ins vs. Ctrl - Experiment 3 - Peptide List
- Supplementary Table S10 (.xls, 912 KB) - PE vs. Ctrl - Experiment 1 - Protein List
- Supplementary Table S11 (.xlsx, 551 KB) - PE vs. Ctrl - Experiment 1 - Peptide List
- Supplementary Table S12 (.xls, 1.4 MB) - PE vs. Ctrl - Experiment 2 - Protein List
- Supplementary Table S14 (.xlsx, 851 KB) - PE vs. Ctrl - Experiment 2 - Peptide List
- Supplementary Table S14 (.xls, 889 KB) - PE vs. Ctrl - Experiment 3 - Protein List
- Supplementary Table S15 (.xlsx, 324 KB) - PE vs. Ctrl - Experiment 3 - Peptide List
- Supplementary Table S16 (.xls, 1.7 MB) - PE vs. Ctrl - Experiment 4 - Protein List
- Supplementary Table S17 (.xlsx, 813 KB) - PE vs. Ctrl - Experiment 4 - Peptide List
- Supplementary Table S18 (.xlsx, 19 KB) - Summary Table of Ins or PE + RAPA vs. Ctrl
- Supplementary Table S19 (.xlsx, 116 KB) - Ins + RAPA vs. Ins - Experiment 1 - Protein List
- Supplementary Table S20 (.xlsx, 1.6 MB) - Ins + RAPA vs. Ins - Experiment 1 - Peptide List
- Supplementary Table S21 (.xlsx, 181 KB) - Ins + RAPA vs. Ins - Experiment 2 - Protein List
- Supplementary Table S22 (.xlsx, 3.5 MB) - Ins + RAPA vs. Ins - Experiment 2 - Peptide List
- Supplementary Table S23 (.xlsx, 190 KB) - Ins + RAPA vs. Ins - Experiment 3 - Protein List
- Supplementary Table S24 (.xlsx, 4.0 MB) - Ins + RAPA vs. Ins - Experiment 3 - Peptide List
- Supplementary Table S25 (.xlsx, 140 KB) - PE + RAPA vs. PE - Experiment 1 - Protein List
- Supplementary Table S26 (.xlsx, 2.3 MB) - PE + RAPA vs. PE - Experiment 1 - Peptide List
- Supplementary Table S27 (.xlsx, 146 KB) - PE + RAPA vs. PE - Experiment 2 - Protein List
- Supplementary Table S28 (.xlsx, 2.5 MB) - PE + RAPA vs. PE - Experiment 2 - Peptide List
- Supplementary Table S29 (.xlsx, 190 KB) - PE + RAPA vs. PE - Experiment 3 - Protein List
- Supplementary Table S30 (.xlsx, 3.8 MB) - PE + RAPA vs. PE - Experiment 3 - Peptide List
- Supplementary Figures Legends (.pdf, 66 KB) - Supplementary Figures Legends
- Supplementary Figure S1 (.pdf, 1.2 MB) - Supplementary Figure S1
- Supplementary Figure S2 (.pdf, 62 KB) - Supplementary Figure S2
- Supplementary Figure S3 (.pdf, 92 KB) - Supplementary Figure S3
- Supplementary Figure S4 (.pdf, 46 KB) - Supplementary Figure S4
- Supplementary Figure S5 (.pdf, 268 KB) - Supplementary Figure S5
- Supplementary Figure S6 (.pdf, 634 KB) - Supplementary Figure S6
