# Supplementary material for: Quantitative Non-canonical Amino Acid Tagging (QuaNCAT) Proteomics Identifies Distinct Patterns of Protein Synthesis Rapidly Induced by Hypertrophic Agents in Cardiomyocytes, Revealing New Aspects of Metabolic Remodeling
Source: Mol Cell Proteomics. 2016 Aug 9;15(10):3170–89. doi: 10.1074/mcp.M115.054312 (PMC5054342; doi:10.1074/mcp.M115.054312)
Supplement: Supplemental Data [file 10.1074_M115.054312_mcp.M115.054312-1.docx]

**Supplemental Table S1. Primers used for 5’-RACE.**

| Gene Name | Primer Function | Primer Sequence |
| --- | --- | --- |
| F-LINKER | Forward primer in PCR round 1 &2 | TGCCACGCTGCTCGCAAACG |
| *Desmin* | Reverse transcription | CGGATGTCCCTGAGGGCGGCTGTGAG |
|  | Nested PCR round1 reverse | ATGGTGCTCGGGTGGTC |
|  | Nested PCR round2 reverse | CGCTGGCTGGACGAGTA |
| *Jup* | Reverse transcription | CAGGTCACTGGTGTTCTGCATGGTCCGC |
|  | Nested PCR round1 reverse | TGCCGCAGGTGTCATCC |
|  | Nested PCR round2 reverse | ATGCCCGAGTCATAGGTGTA |
| *Aldoa* | Reverse transcription | TCAGACAGCCCGTCCAGCCCTTGAGTAG |
|  | Nested PCR round1 reverse | TTCTCCTCGGTGTTCTCG |
|  | Nested PCR round2 reverse | GGTCAGTGCTGGGTATGG |
