# Supplementary material for: Quantitative Non-canonical Amino Acid Tagging (QuaNCAT) Proteomics Identifies Distinct Patterns of Protein Synthesis Rapidly Induced by Hypertrophic Agents in Cardiomyocytes, Revealing New Aspects of Metabolic Remodeling
Source: Mol Cell Proteomics. 2016 Aug 9;15(10):3170–89. doi: 10.1074/mcp.M115.054312 (PMC5054342; doi:10.1074/mcp.M115.054312)
Supplement: Supplemental Data [file 10.1074_M115.054312_mcp.M115.054312-2.docx]

**Supplemental Table S2. Primers used for qPCR.**

| Species | Gene Name | Primer Name | Primer Sequence |
| --- | --- | --- | --- |
| Mice | *Mdh2* | Forward | GTCAGAGCGAACACGTTTGT |
|  |  | Reverse | GGGGTACACTGAGAGATCAGG |
|  | *Jup* | Forward | GGAGCTCACCAATTCCCTCT |
|  |  | Reverse | TAGGTGGCATCCATGTCGTC |
|  | *Aco2* | Forward | ATCTGATTGAGGCCCAGGTT |
|  |  | Reverse | GAATGATCTGGTGAATGATCCCA |
|  | *Aldoa* | Forward | TCAAGTCCAAGGGTGGTGTT |
|  |  | Reverse | ATCCAGCCCCTGGGTAGTT |
|  | *Hsp60* | Forward | AAAATTTGGTGCGGACGCT |
|  |  | Reverse | TTGGGACTTCCCCAACTCTG |
|  | *Rps6* | Forward | TGGTGAAGAGTGGAAGGGTT |
|  |  | Reverse | CCAGTTCTCCTTGGCCTGTA |
| Rat | *Anf* | Forward | GCAGGCCCTGAGCGAGC |
|  |  | Reverse | CTTCGGTACCGGAAGCTGT |
|  | *Bnp* | Forward | CCGCTGGGAGGTCACTCCCAT |
|  |  | Reverse | ACAGCCCAAGCGACTGACTGC |
|  | *Aco2* | Forward | TCAATGAGCTAAAGCCACATATCA |
|  |  | Reverse | TCGTAGCTTGAATTGGTGCAG |
|  | *Hsp60* | Forward | GCTGTAGCTGTTACAATGGGG |
|  |  | Reverse | CTGAACAAGCTTAGCTCCGAT |
|  | *Pkm1/2* | Forward | TCATCTGTACCATTGGCCCT |
|  |  | Reverse | GCTGCACGGACATTCTTGAT |
|  | *Mb* | Forward | TGCTGAACATCTGGGGGAAA |
|  |  | Reverse | GGGGTGAGCCTTAAATAGACTGA |
|  | *Desmin* | Forward | TGACTCAGGCAGCCAATAAG |
|  |  | Reverse | CTCATCTGCCTCATCAAGGA |
|  | *Acadl* | Forward | AGAGCTGATCGCAAGACAGA |
|  |  | Reverse | CCCTTCCAGGTTCCACTTTA |
|  | *Eef2* | Forward | TCCCAACATTCTCACCGACA |
|  |  | Reverse | AAGAGCACCCTCCTTAGTGG |
|  | *Eif4e* | Forward | GCAAACCTTCGGTTGATCTCT |
|  |  | Reverse | CCACATAGGCTCAATACCATCCTTA |
|  | *Eif1g* | Forward | TGAGGAAGAGATGGATGAGTGT |
|  |  | Reverse | TCCTCATTGGAGTACTTTCGCT |
