# Supplementary material for: Quantitative Non-canonical Amino Acid Tagging (QuaNCAT) Proteomics Identifies Distinct Patterns of Protein Synthesis Rapidly Induced by Hypertrophic Agents in Cardiomyocytes, Revealing New Aspects of Metabolic Remodeling
Source: Mol Cell Proteomics. 2016 Aug 9;15(10):3170–89. doi: 10.1074/mcp.M115.054312 (PMC5054342; doi:10.1074/mcp.M115.054312)
Supplement: Supplemental Data [file 10.1074_M115.054312_mcp.M115.054312-31.pdf]

Supplemental Figure Legends

Quantitative Non-Canonical Amino acid Tagging based proteomics identifies distinct patterns of protein synthesis rapidly induced by hypertrophic agents in cardiomyocytes, revealing new aspects of metabolic remodeling

Supplemental Figure S1. Insulin increases the cell size of ARVC and the levels of the mRNA for ANF/BNP. (A) ARVC were treated with 100 nmol/L insulin for 48 h, cells were then labelled with Cellmask stain and then imaged for estimation of cell size (scale bar = 50 μm). Cellmask: red; DAPI (for nuclei stain): blue. (B) Quantification of A. Data are presented as means ± SD (n = 10). **, $0.001<P<0.01$ as determined by unpaired student's t-test. (C) ARVC were treated with insulin (10nmol/L) for 48 hours prior to cell lysis. Levels of ANF and BNP mRNA were quantified by RT-qPCR. Data are normalized to total 18S rRNA.

Supplemental Figure S2. Scheme of click reaction on solid surface (nitrocellulose membrane), See EXPERIMENTAL PROCEDURES for further information

Supplemental Figure S3. The expression of PTBP1 and PKM1 are not changed in ARVC or rat hearts under hypertrophic stimulation. (A) ARVC were cultured and treated as described in main Figure. 5C, after lysis, 20μg of total proteins were subjected to western

blot. (B,C) Control and TAC rats were described in Figure.5A, Left ventricles were homogenised in liquid nitrogen, then lysed with RIPA buffer, and protein concentration was measured by the BCA method. 20 μg of protein from each heart was subjected to western blot analysis using the indicated antibodies.

Supplemental Figure S4. Scheme of modified method for rapid amplification of cDNA ends (RACE), See EXPERIMENTAL PROCEDURES for further information

Supplemental Figure S5. PCR result for 5'-RACE. (A) First strand cDNA was synthesized from 1μg total RNA from rat heart by using Desmin gene specific primer, then 1pmol or 10pmol of polynucleotide linker were ligated with the first strand cDNA by T4 RNA ligase. After the ligation reaction, nested PCR was conducted. The primers for the first round PCR were F and RT. Primers for the second round PCR were as indicated. Template for the first round PCR was the ligation product and the template for the second round PCR was the product of the first round PCR. Products of the second round PCR were analysed by agarose electrophoresis. The indicated PCR product was recovered from agarose gel and sent for Sanger sequencing. (B,C)RT reaction, ligation reaction(10pmol of polynucleotide

linker were used) and nested PCR was described in panel A. Primers for the first round PCR were as indicated, Products of both rounds of PCR were analysed by agarose electrophoresis. F=Foward primer, which was complementary to the polynucleotide linker.

Supplemental Figure S6. The expression of PKM2 and eEF1G are not changed in pregnancy or exercise induced CH. (A) Exercise training was as described in EXPERIMENTAL PROCEDURES. At 10-week time point, seven experimental rats and six age-paired controls were sacrificed. Total protein was extracted from the left ventricles and analysed by Western blot. (B). Mating mice were set up as described in EXPERIMENTAL PROCEDURES. Pregnant mice were sacrificed at the indicated times post-insemination, then total protein was extracted from the left ventricles and analysed by Western blot.
