# Supplementary figures and images for: Quantitative Non-canonical Amino Acid Tagging (QuaNCAT) Proteomics Identifies Distinct Patterns of Protein Synthesis Rapidly Induced by Hypertrophic Agents in Cardiomyocytes, Revealing New Aspects of Metabolic Remodeling
Source: Mol Cell Proteomics. 2016 Aug 9;15(10):3170–89. doi: 10.1074/mcp.M115.054312 (PMC5054342; doi:10.1074/mcp.M115.054312)

Supplemental Figure S1

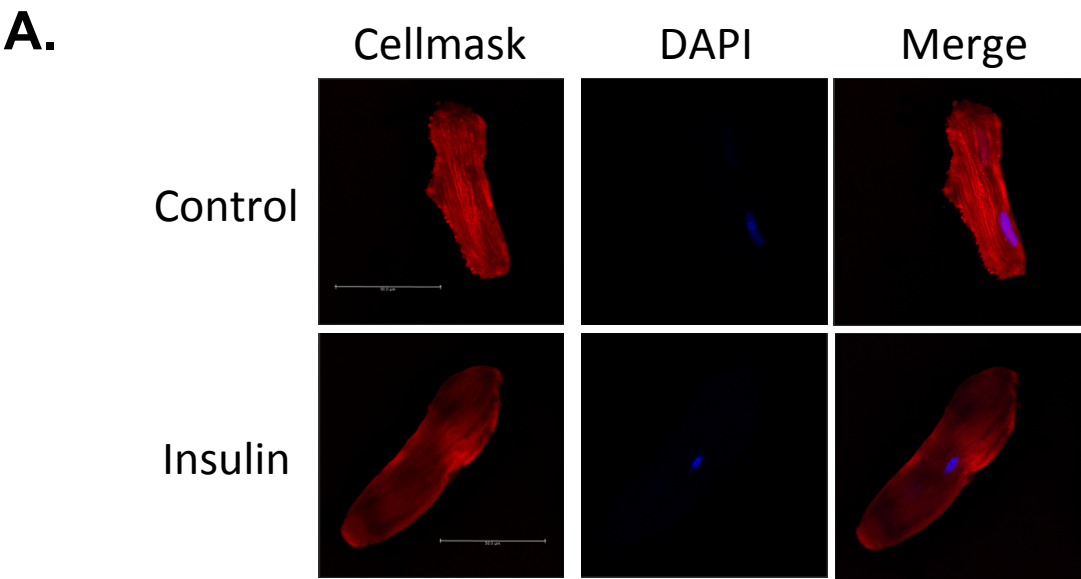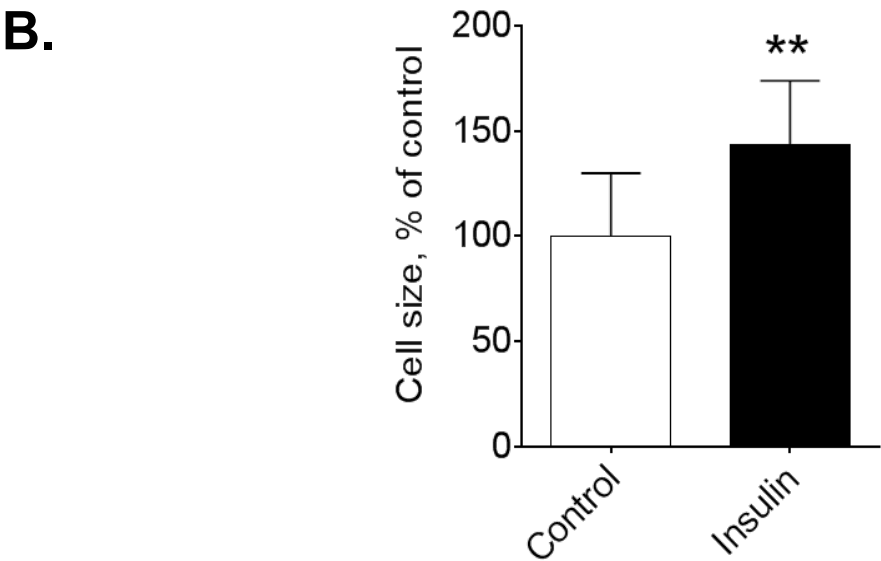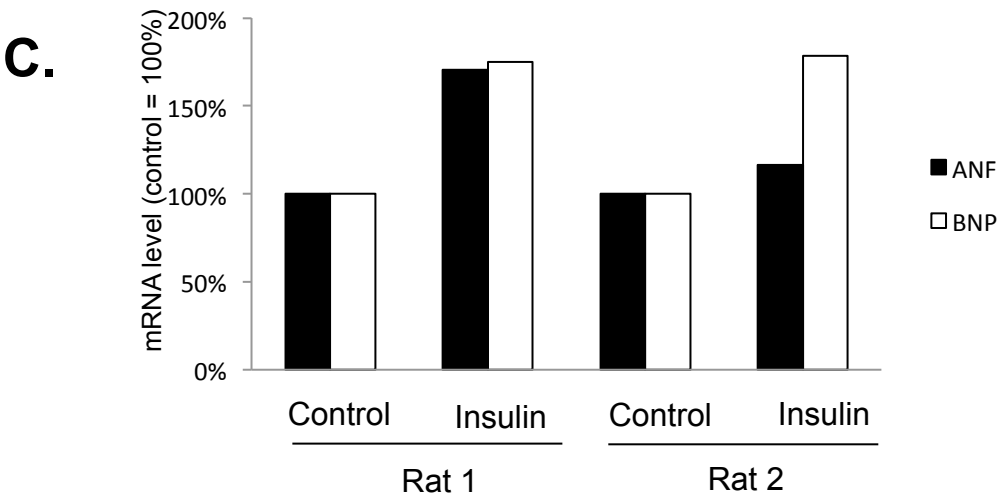

Supplement: Supplemental Data [file 10.1074_M115.054312_mcp.M115.054312-32.pdf]

# Supplemental Figures

Supplemental Figure S2

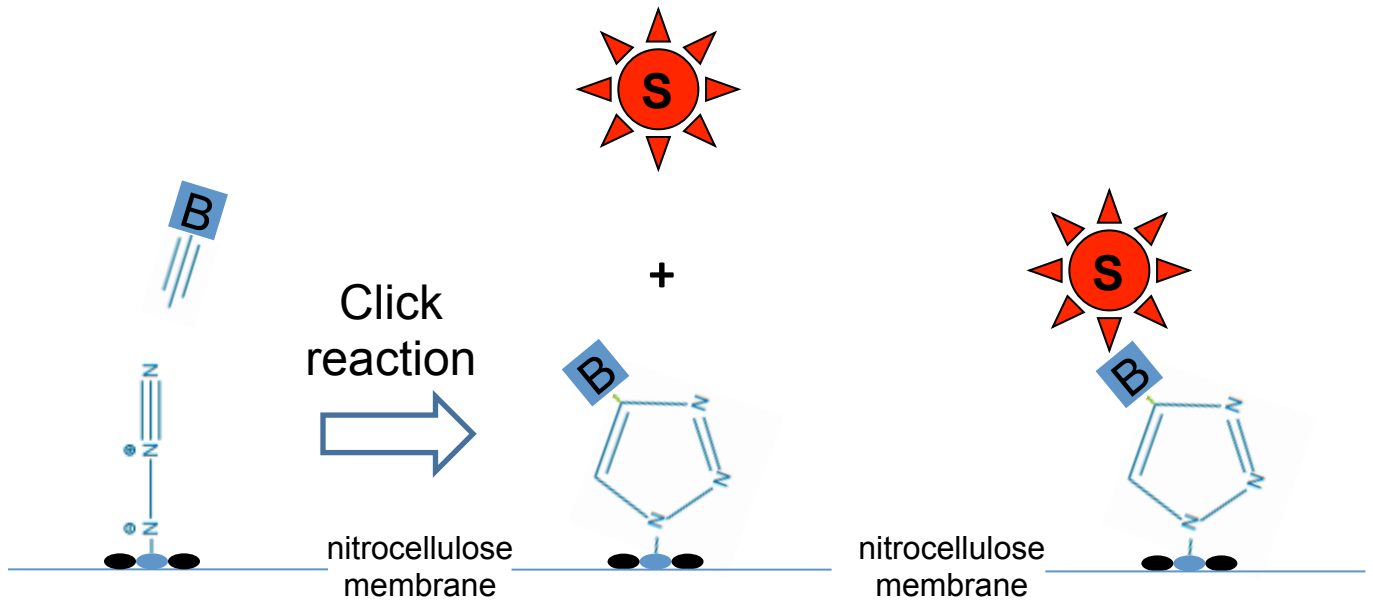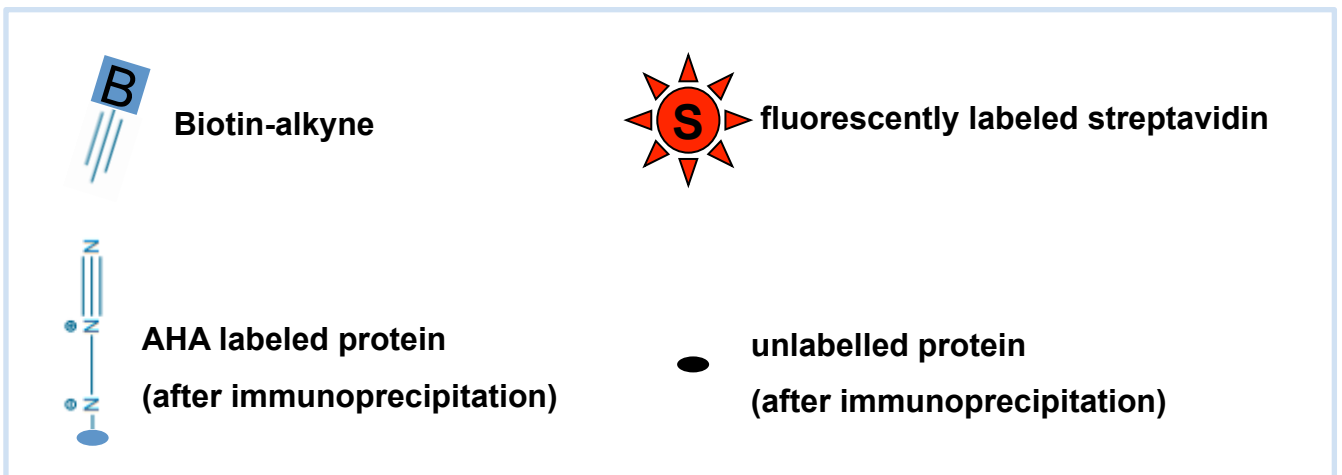

Supplement: Supplemental Data [file 10.1074_M115.054312_mcp.M115.054312-33.pdf]

Supplemental Figure S3

**A.**

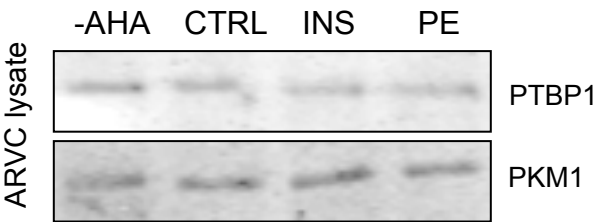

**B.**

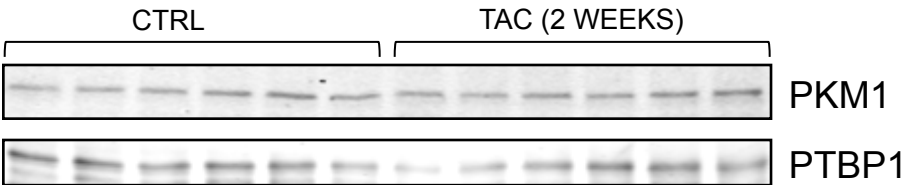

**C.**

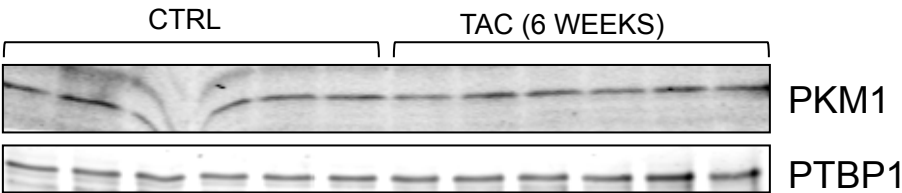

Supplement: Supplemental Data [file 10.1074_M115.054312_mcp.M115.054312-34.pdf]

Supplemental Figure S4

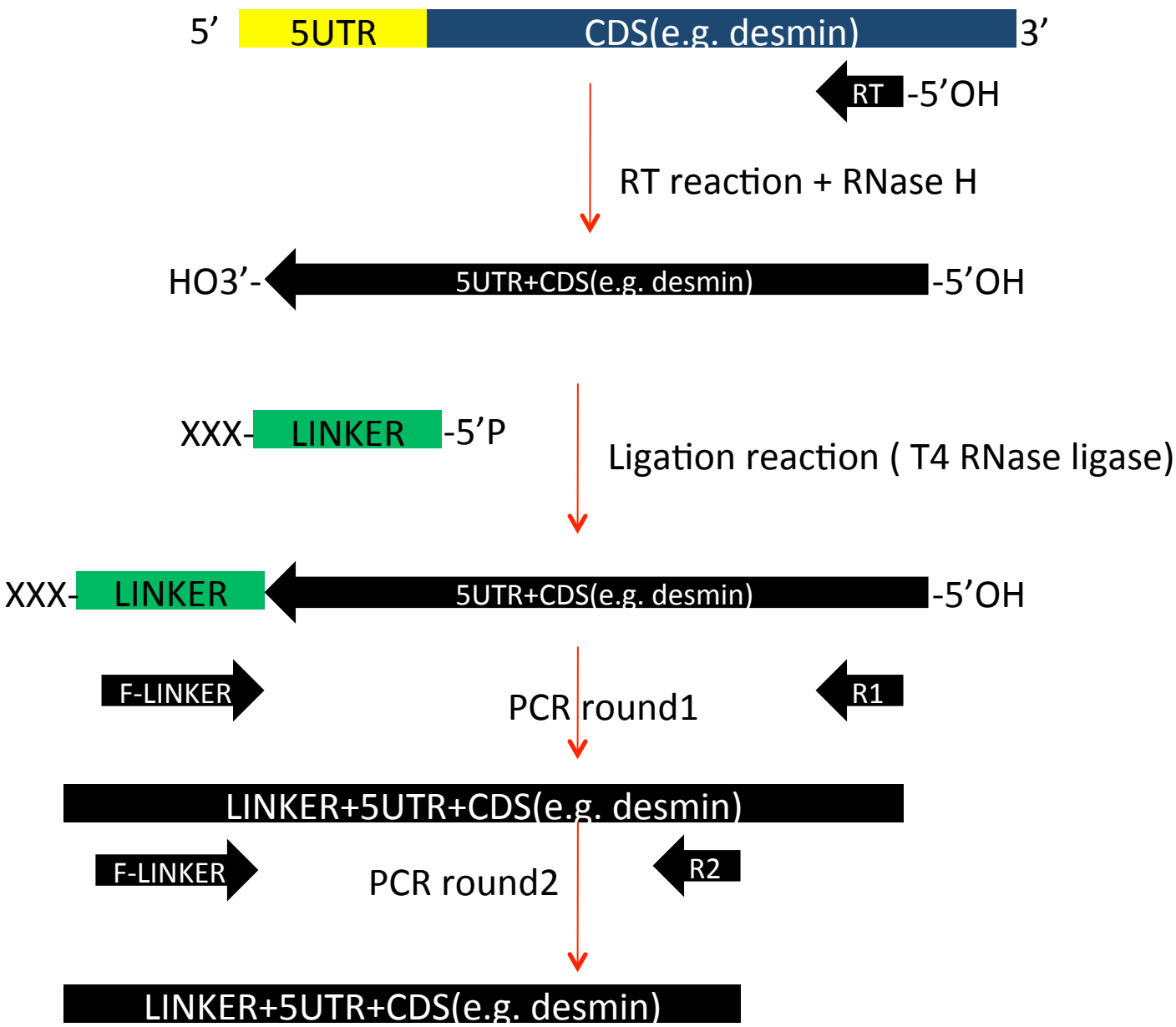

Supplement: Supplemental Data [file 10.1074_M115.054312_mcp.M115.054312-35.pdf]

Supplemental Figure S5

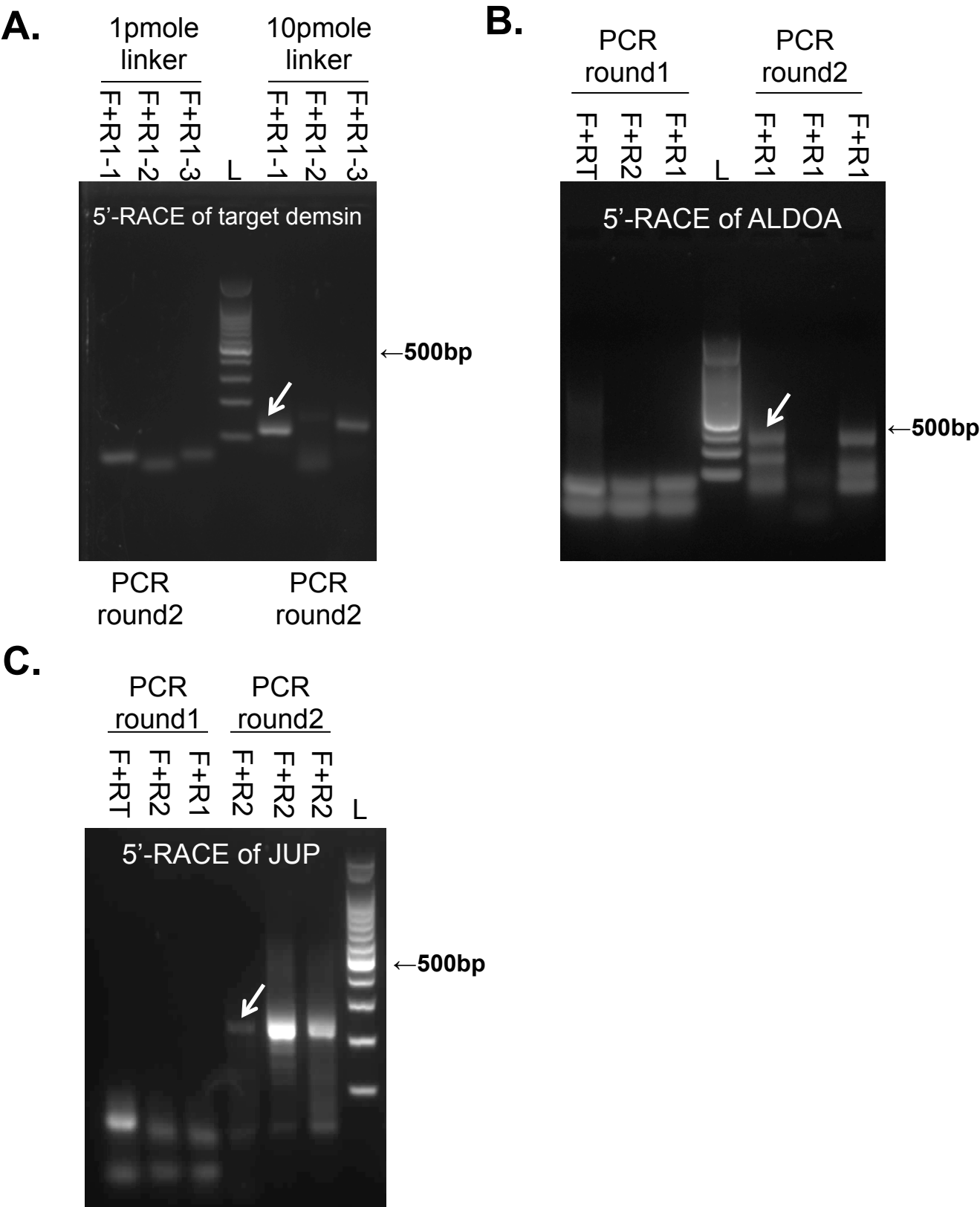

Supplement: Supplemental Data [file 10.1074_M115.054312_mcp.M115.054312-36.pdf]

**A.**

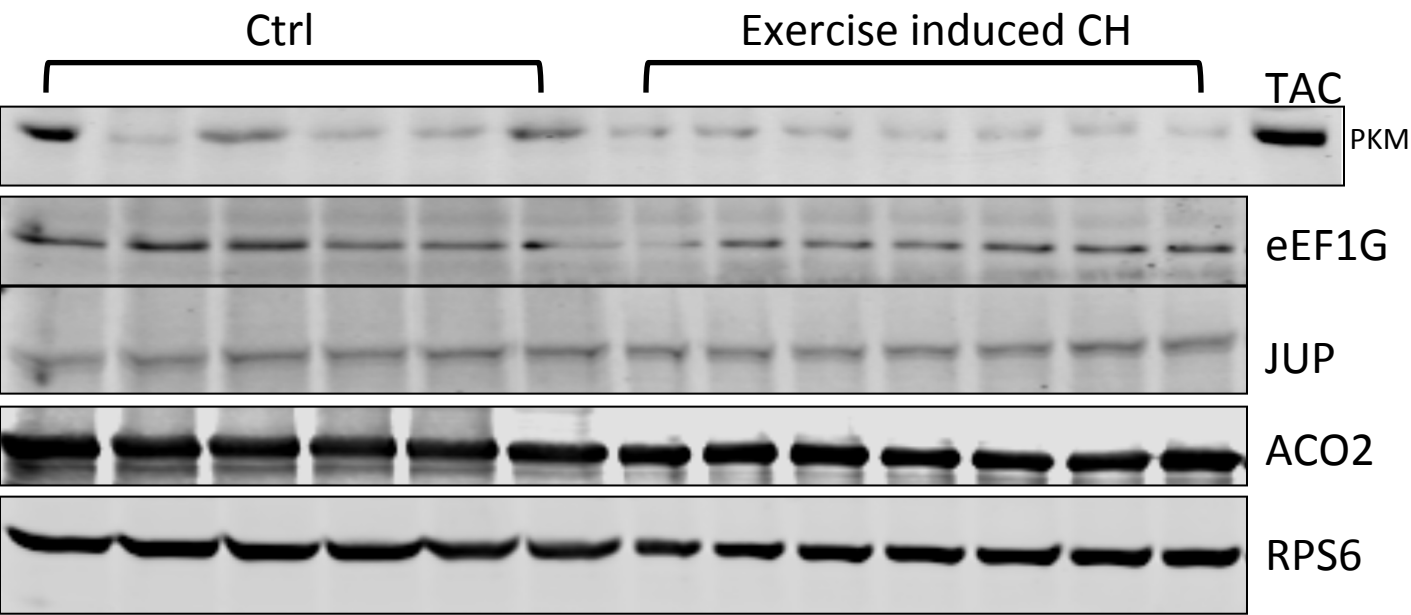

**B.**

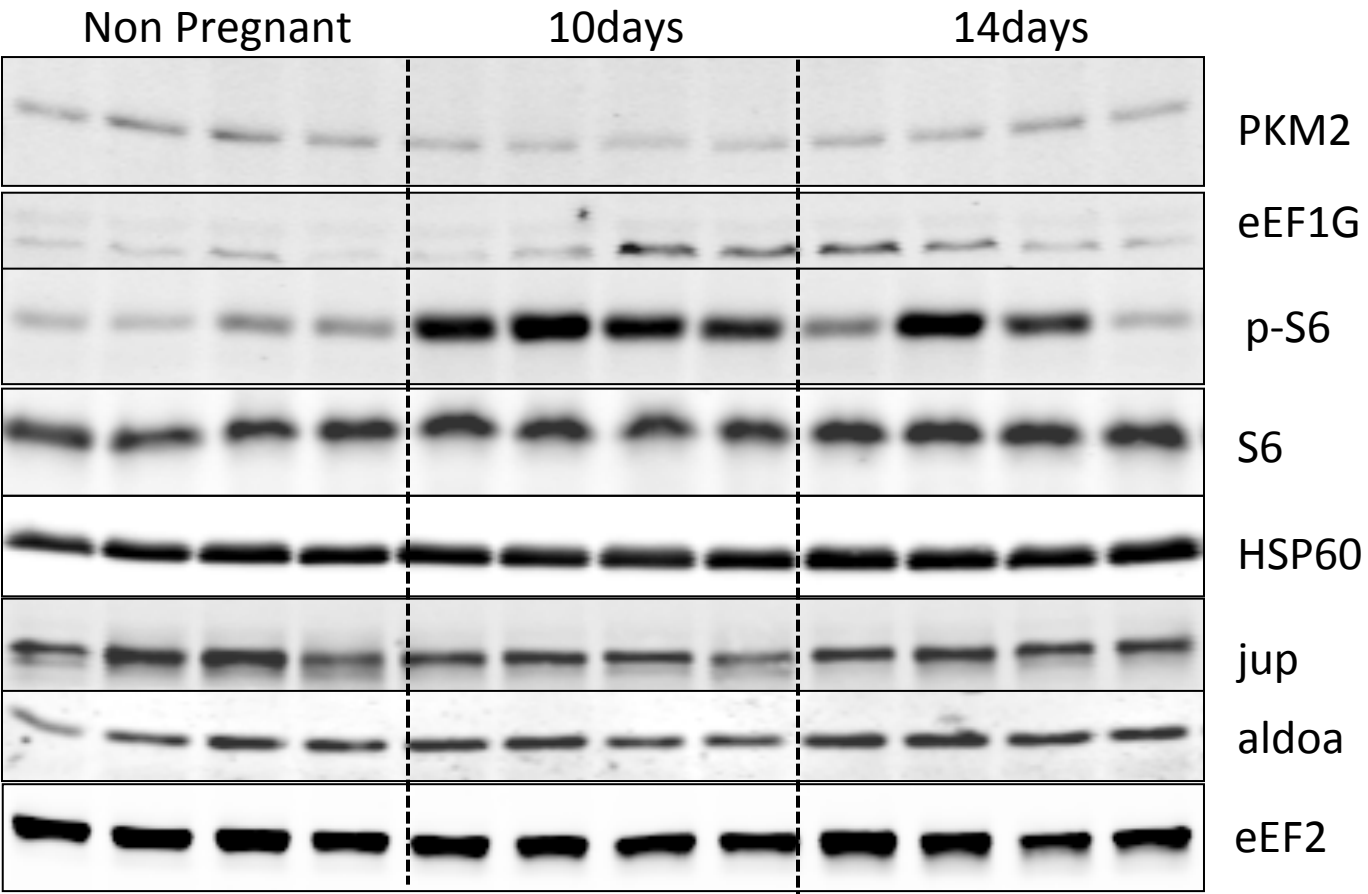

Supplement: Supplemental Data [file 10.1074_M115.054312_mcp.M115.054312-37.pdf]
